# Surveying brain tumor heterogeneity by single-cell RNA-sequencing of multi-sector biopsies

**DOI:** 10.1093/nsr/nwaa099

**Published:** 2020-05-30

**Authors:** Kai Yu, Yuqiong Hu, Fan Wu, Qiufang Guo, Zenghui Qian, Waner Hu, Jing Chen, Kuanyu Wang, Xiaoying Fan, Xinglong Wu, John EJ Rasko, Xiaolong Fan, Antonio Iavarone, Tao Jiang, Fuchou Tang, Xiao-Dong Su

**Affiliations:** Biomedical Pioneering Innovation Center (BIOPIC), School of Life Sciences, Peking University, Beijing 100871, China; Biomedical Pioneering Innovation Center (BIOPIC), School of Life Sciences, Peking University, Beijing 100871, China; Beijing Advanced Innovation Center for Genomics, School of Life Sciences, Peking University, Beijing 100871, China; Biomedical Institute for Pioneering Investigation via Convergence and Center for Reproductive Medicine, Ministry of Education Key Laboratory of Cell Proliferation and Differentiation, Beijing 100871, China; Department of Molecular Neuropathology, Beijing Neurosurgical Institute, Capital Medical University, Beijing 100050, China; Department of Neurosurgery, Beijing Tiantan Hospital, Capital Medical University, Beijing 100050, China; Chinese Glioma Genome Atlas Network (CGGA) and Asian Glioma Genome Atlas Network (AGGA), Beijing 100050, China; Biomedical Pioneering Innovation Center (BIOPIC), School of Life Sciences, Peking University, Beijing 100871, China; Department of Neurosurgery, Beijing Tiantan Hospital, Capital Medical University, Beijing 100050, China; Biomedical Pioneering Innovation Center (BIOPIC), School of Life Sciences, Peking University, Beijing 100871, China; Department of Molecular Neuropathology, Beijing Neurosurgical Institute, Capital Medical University, Beijing 100050, China; Department of Neurosurgery, Beijing Tiantan Hospital, Capital Medical University, Beijing 100050, China; Chinese Glioma Genome Atlas Network (CGGA) and Asian Glioma Genome Atlas Network (AGGA), Beijing 100050, China; Department of Molecular Neuropathology, Beijing Neurosurgical Institute, Capital Medical University, Beijing 100050, China; Department of Neurosurgery, Beijing Tiantan Hospital, Capital Medical University, Beijing 100050, China; Chinese Glioma Genome Atlas Network (CGGA) and Asian Glioma Genome Atlas Network (AGGA), Beijing 100050, China; Biomedical Pioneering Innovation Center (BIOPIC), School of Life Sciences, Peking University, Beijing 100871, China; Beijing Advanced Innovation Center for Genomics, School of Life Sciences, Peking University, Beijing 100871, China; Biomedical Institute for Pioneering Investigation via Convergence and Center for Reproductive Medicine, Ministry of Education Key Laboratory of Cell Proliferation and Differentiation, Beijing 100871, China; Beijing Advanced Innovation Center for Genomics, School of Life Sciences, Peking University, Beijing 100871, China; Biomedical Institute for Pioneering Investigation via Convergence and Center for Reproductive Medicine, Ministry of Education Key Laboratory of Cell Proliferation and Differentiation, Beijing 100871, China; Peking-Tsinghua Center for Life Sciences, Peking University, Beijing 100871, China; Gene and Stem Cell Therapy Program, Centenary Institute, University of Sydney, Sydney, NSW, Australia; Department of Cell and Molecular Therapies, Royal Prince Alfred Hospital, Sydney, NSW, Australia; Beijing Key Laboratory of Gene Resource and Molecular Development, Laboratory of Neuroscience and Brain Development, Beijing Normal University, Beijing 100875, China; Institute for Cancer Genetics, Herbert Irving Comprehensive Cancer Center, Columbia University Medical Center, New York, NY 10032, USA; Department of Pathology & Cell Biology, Columbia University Medical Center, New York, NY 10032, USA; Department of Neurology, Columbia University Medical Center, New York, NY 10032, USA; Department of Molecular Neuropathology, Beijing Neurosurgical Institute, Capital Medical University, Beijing 100050, China; Department of Neurosurgery, Beijing Tiantan Hospital, Capital Medical University, Beijing 100050, China; Chinese Glioma Genome Atlas Network (CGGA) and Asian Glioma Genome Atlas Network (AGGA), Beijing 100050, China; Center of Brain Tumor, Beijing Institute for Brain Disorders, Beijing 100069, China; Biomedical Pioneering Innovation Center (BIOPIC), School of Life Sciences, Peking University, Beijing 100871, China; Beijing Advanced Innovation Center for Genomics, School of Life Sciences, Peking University, Beijing 100871, China; Biomedical Institute for Pioneering Investigation via Convergence and Center for Reproductive Medicine, Ministry of Education Key Laboratory of Cell Proliferation and Differentiation, Beijing 100871, China; Peking-Tsinghua Center for Life Sciences, Peking University, Beijing 100871, China; Biomedical Pioneering Innovation Center (BIOPIC), School of Life Sciences, Peking University, Beijing 100871, China

**Keywords:** glioma, heterogeneity, multi-sector biopsy, single-cell RNA-seq

## Abstract

Brain tumors are among the most challenging human tumors for which the mechanisms driving progression and heterogeneity remain poorly understood. We combined single-cell RNA-seq with multi-sector biopsies to sample and analyze single-cell expression profiles of gliomas from 13 Chinese patients. After classifying individual cells, we generated a spatial and temporal landscape of glioma that revealed the patterns of invasion between the different sub-regions of gliomas. We also used single-cell inferred copy number variations and pseudotime trajectories to inform on the crucial branches that dominate tumor progression. The dynamic cell components of the multi-region biopsy analysis allowed us to spatially deconvolute with unprecedented accuracy the transcriptomic features of the core and those of the periphery of glioma at single-cell level. Through this rich and geographically detailed dataset, we were also able to characterize and construct the chemokine and chemokine receptor interactions that exist among different tumor and non-tumor cells. This study provides the first spatial-level analysis of the cellular states that characterize human gliomas. It also presents an initial molecular map of the cross-talks between glioma cells and the surrounding microenvironment with single-cell resolution.

## INTRODUCTION

Gliomas are among the most lethal forms of human tumors as they are characterized by aggressive behaviors and resistance to multiple therapies. The development of genetic mutations in malignant cells and the complex interactions between tumor and non-tumor cells in the glioma microenvironment foster intratumoral heterogeneity, thus contributing to therapeutic failures and the generally poor prognosis of gliomas. A major unmet challenge in neuro-oncology is our ability to understand glioma heterogeneity and progression in gliomas and how they influence therapeutic resistance [1].

Several studies have reported that malignant gliomas are characterized by a formidable degree of intratumoral heterogeneity. For example, mosaic amplification of receptor tyrosine kinase genes (*EGFR*, *MET*, *PDGFRA*) is known to represent a classical hallmark of genetic heterogeneity affecting neighboring tumor cells within bulk glioma samples [2]. Furthermore, single cell-derived clones of glioma cells have been identified and shown to exhibit divergent proliferation and differentiation abilities [3]. Finally, the multi-region genetic analysis of gliomas with single nucleotide polymorphism (SNP) array or whole exome sequencing has revealed that divergent glioma subtypes can be recovered from different geographical regions, which together give rise to a branched pattern of progression [4,5]. As single-cell RNA-sequencing became a feasible approach to investigate human tumors, glioma heterogeneity has started to be explored with single-cell resolution [6–11]. However, most of the studies that have previously reported single-cell RNA-sequencing of gliomas did not include analysis of either tumor cells and the tumor microenvironment (TME) from multiple spatially annotated regions of gliomas, thus limiting our understanding of patterns of spatial evolution and brain infiltration, the latter being one of the most critical hallmarks of aggressiveness and progression of malignant glioma.

To delineate the glioma single-cell heterogeneity in both spatial and temporal resolution, we performed a glioma single-cell analysis from multi-sector biopsies informed by precision navigation surgery. Cell type components of each tumor fragment and temporal relationship of cells in each individual patient were unbiasedly identified. Our analysis did not use approaches aimed at selecting specific tumor or non-tumor cell populations. Therefore, we report the first single-cell-based comprehensive spatial analysis of the geographical structure of glioma and the dynamic progression of the interactions of tumor cells with individual non-tumor cells from multiple tumor locations.

## RESULTS

### Precision navigation-based multi-sector biopsies and single-cell RNA-seq of glioma cells

**Figure 1.**
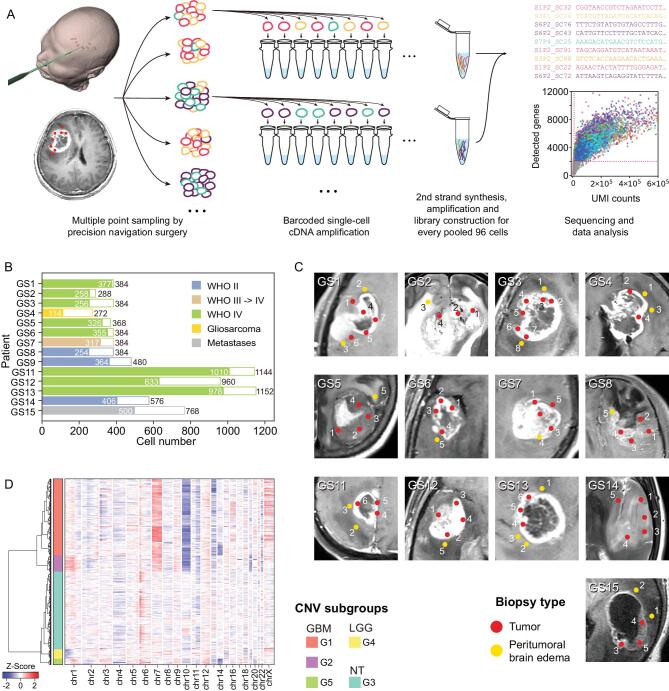
General information on experimental procedure and generated data. (A) Experimental procedure. Multiple point sampling was done by precision navigation surgery, followed by single-cell isolation and barcoded single-cell cDNA amplification. After every 96 cells were pooled together, the sequencing library was constructed by several experimental procedures. (B) Pathology information and cell number of 14 patients collected in this study. White bars and colored bars represent raw cell number and filtered cell number in each patient, respectively. (C) Medical imaging and biopsy locations of all sampling points in each patient. Red and yellow dots mark locations of tumoral and peritumoral sampling points in the MRI image. (D) RNA-derived single-cell CNV information. Hierarchical clustering divided all glioma-related cells into five CNV subtypes.

Tumor sections with potential representative divergent properties were marked in a presurgical 3D enhanced magnetic resonance imaging (MRI) model and tumor tissues were precisely collected during surgery by navigation sampling. Samples were quickly dissociated and subjected to single-cell RNA-sequencing (scRNA-seq) library preparation [12,13] (Fig. 1A). Overall, 7928 single-cell transcriptomes were generated, and 6148 passed stringent quality filtering steps after alignment and reads counting (Fig. 1B and Supplementary Fig. S1A,B). These cells were collected from 73 regions in 13 patients with glioma covering the most frequent subtypes (3 WHO grade II, 1 WHO grade III, 8 WHO grade IV and 1 gliosarcoma). As a control, we also included one brain metastasis from a patient with lung squamous cell carcinoma (Fig. 1C, Supplementary Fig. S2, Supplementary Tables 1 and 2).

As regional gene expression status can be affected by copy number variation (CNV) dose effect, we adopted previously reported methods to predict large fragment copy number status with a single-cell gene expression matrix [6,8]. The generated copy number matrix clustered into five CNV subgroups (Fig. 1D). Malignant cells were identified based on this classification. Subgroups G1 and G2 included glioblastoma multiforme (GBM) cells sharing a *chr7^amp^*/*chr10^del^*-driven transcriptome, whereas a unique CNV pattern was apparent in GBM patient GS3 (subgroup G5). The G4 subgroup included low-grade glioma (LGG) cells with *chr1p*/*chr19q* codeletion-driven signatures. The last subgroup (G3) was composed of non-malignant cells without obvious CNVs.

### Optimized t-SNE map and clustering identifies 25 cell type clusters

As reported by previous single-cell RNA-seq studies of human glioma, the malignant cells from different patients showed a fragmented relationship in clustering analysis, mainly because of the dose effect of gene expression caused by diverse CNVs [6,8]. Therefore, we argued that removal of CNV variances would optimize the principal component analysis (PCA) and t-Distributed Stochastic Neighbor Embedding (t-SNE) analysis.

**Figure 2.**
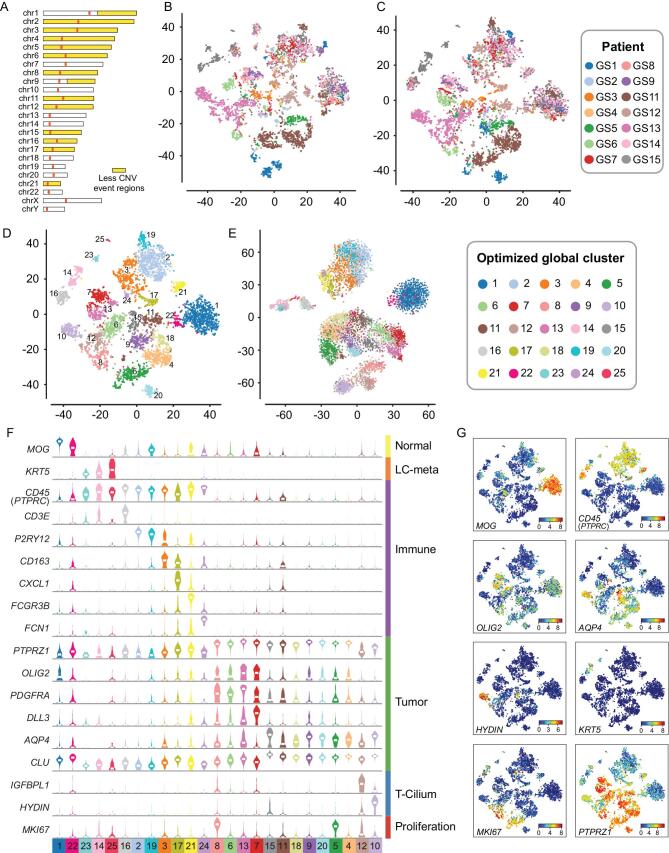
Molecular characteristics of cells from glioma. (A) Based on bulk CNV status, chromosome regions that had fewer CNV events (1.8<copy<2.2, abnormal patient<3) in our samples were marked by yellow color, and variable genes in these regions were used for PCA and t-SNE analysis. (B) Pre-optimized global t-SNE map, colored by patients. (C) After-optimized global t-SNE map, colored by patients. (D) Global map of sub-clusters using t-SNE coordinates. Dots are individual cells, colored by optimized global clusters. (E) t-SNE analysis based on binary regulon activity, analyzed by SCENIC. Clusters had similar distribution with the global 25 cluster t-SNE map. (F) was constructed according to (D). With shared and specifically expressed patterns as shown in (G), sub-clusters could be divided into normal (*MOG*^+^), immune (*CD45*^+^), regular glioma tumor (*PTPRZ1*^+^, *OLIG2*^+^ or *AQP4*^+^), cilia property tumor (*HYDIN*^+^), proliferation tumor (*MKI67*^+^) and metastasis cells.

To obtain the CNV status of each tumor sample, we performed low-depth whole genome sequencing (WGS) on bulk biopsies. Based on the WGS data, genes with interpatient large copy number changes were removed from the clustering analysis (Fig. 2A, Supplementary Figs S3B and S4). The integrated analysis of tumor cells from our entire dataset using PCA and t-SNE resulted in an optimized map of 25 cell clusters (Fig. 2C, D and Supplementary Fig. S5). Compared to the original t-SNE map (Fig. 2B and Supplementary Fig. S6A, B and D), the fragmented cell distribution was resolved to a map with more concentrated points and clearer edges (Fig. 2D). This distribution was confirmed by independent binary regulon activity clustering with SCENIC (Fig. 2E and Supplementary Figs S6E and S7) [14]. A combination of these different strategies could help minimize analyze artifacts in analysis.

Considering the cell source and CNV status with a global t-SNE map, the non-tumor cells and the glioma cells lacking extensive genomic rearrangements (such as those derived from LGG) showed a converged distribution, indicating that our data were not influenced by batch effects. They also indicated that the LGG-derived tumor cells were more uniform among different patients (Fig. 2C and Supplementary Fig. S6C). Cell cluster-specific genes coalesced the 25 clusters into four major groups (Supplementary Table 2): *CNV^−^MOG^+^* normal glial cells [15], *KRT5^+^* lung cancer (LC) metastasis cells [16], *CD45* (*PTPRC*)^+^ immune cells and *CNV*^+^ malignant tumor cells. In malignant glioma cells, *OLIG2*/*DLL3* and *AQP4*/*CLU* distinguish tumor cells exhibiting transcriptomic features of oligodendrocytes and astrocytes, respectively [17–19]. Cells positive to the general proliferative marker *MKI67*^+^ were present among both glioma cell phenotypes. Moreover, *PTPRZ1* and *SOX2* were significantly overexpressed in glioma cells and could therefore be considered as useful markers to estimate the tumor cell purity in bulk glioma tissues. Interestingly, clusters 10 and 12 specifically overexpressed cilium-related markers (*HYDIN*, *FOXJ1*, etc.) [20,21]. The tumor cells included in these clusters exhibited astrocyte-like features and expressed low levels of *PTPRZ1* (Fig. 2F, G and Supplementary Fig. S8). Clusters 10 and 12 mostly derived from patient GS13, indicated that this patient developed a rare form of ciliated glioma for which single-cell studies have not previously been reported.

### Annotation of the glioma microenvironment and TCGA-based classification of single glioma cells

**Figure 3.**
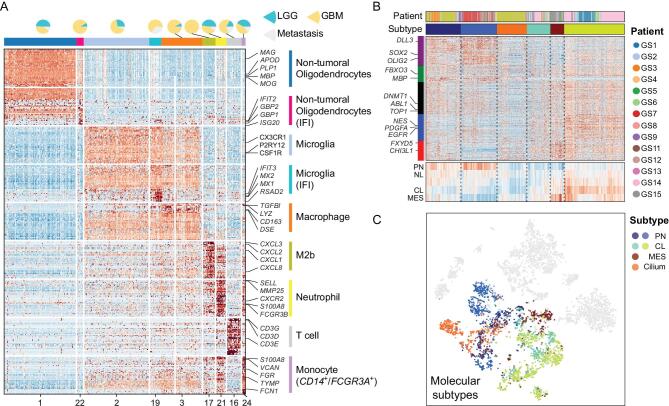
Cell type annotation of tumor microenvironment cells and malignant cells. (A) Heatmap shows marker genes of sub-clusters of tumor microenvironment cells. Pie plot at the top shows cell ratio of LGG/GBM/metastasis samples. Cells in clusters marked with IFI show interferon-induced property. (B) Cells clustered by TCGA 4 classification genes. Heatmap at the bottom shows the average expression value of five gene-sets marked in the left color bar. (C) Tumor cell subtype mapped in t-SNE coordinates.

Normal diploid cells in the tumor samples were mainly cells within the tumor TME. To conduct an unbiased investigation of the TME in glioma, we did not include flow cytometry-based selection in our sampling procedure. Normal oligodendrocytes, microglia and macrophages accounted for approximately half of these cells, with relative homogeneity among patients. Such a conclusion could not have emerged from *CD45* selected samples, as this is the prevailing sampling method used in previous scRNA-seq studies. We also captured cells showing specialized activities, such as interferon-induced oligodendrocytes and polarized microglia/macrophages (Fig. 3A).

Next, we classified malignant cells according to the Cancer Genome Atlas (TCGA) GBM classification model [19]. Grade II tumor cells with chromosome *1p*/*19q* codeletion had a strong proneural (PN) signature. Conversely, the individual tumor cells recovered from GBM were distributed among each of the three transcriptomic subgroups (PN, classical (CL) or mesenchymal (MES)). We did not identify glioma cells that could be assigned to the neural (NL) subtype but we detected cells expressing cilium-related signatures that were distinct from those associated with known TCGA subgroups (Fig. 3B and C).

### CNV accumulation and tumor progression in patient GS1

As CNVs usually accumulate during tumor progression, the CNV profile of individual tumor cells has emerged as an accurate inference to trace tumor progression [2–4,8,22,23]. We used arm-level scRNA-seq-derived CNVs to trace tumor cell clones, which were confirmed by bulk WGS (Fig. 1D, Supplementary Fig. S3A and B) to avoid possible mistakes by unravelling single-cell CNV from RNA data with bioinformatics tools alone. Multiple cell subpopulations with accumulated CNVs were found in patients GS1, GS13 and GS5 (Supplementary Figs S3C and S9).

**Figure 4.**
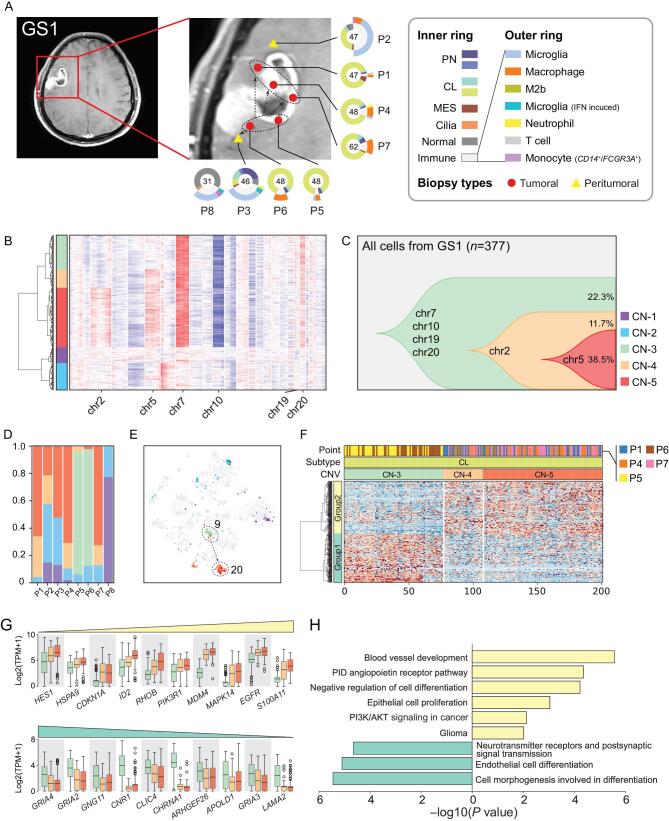
Glioma clonal evolution of Patient GS1 at spatial and temporal resolution. (A) MRI image of Patient GS1. Yellow and red markers in zoomed image represent peritumoral and tumoral sampling points. Ring plot in the right and bottom displays cell components of each point. Color of the inner ring shows classified glial cell subtypes, and the outer ring shows detailed immune cell subtypes. Cell numbers are labeled in the center of these ring plots. (B) Single-cell CNV heatmap. Cells were divided into five groups by hierarchical clustering. (C) Clonal evolution trail followed by accumulating CNV events. Each color represents a CNV subclone and chromosomes are labeled in which copy number alteration occurred during clonal transition. (D) CNV subclone components in each sampling point, P5/6 had a different component compared with other tumoral/peritumoral points. (E) CNV subclone distributions in t-SNE coordinates. When CN-3 developed into CN-4/5, cells translocated from cluster 9 to cluster 20. (F) Heatmap of differentially expressed genes between CN-3/4/5 CL glial cells. Differential genes could be divided into two gene-sets, marked with the left color bar. (G) Box plot of differential genes. The increased (upper) and decreased (lower) gene expression profiles followed CNV accumulation. Boxes were colored by CNV subgroup. (H) Functional enrichment of increased and decreased gene-sets.

From patient GS1, a female with an *IDH*-wild type GBM, single cells were collected from five tumor core locations (P1/4/5/6/7) and two peritumoral locations (P2/3). A few cells (P8) were also collected from an adjacent brain region, which, from the imaging analysis, lacked evidence of tumor infiltration. Ring plots illustrate the tumor and non-tumor cell components at each site (Fig. 4A). In this patient, CL glioma cells accounted for most of the malignant components at all tumor sites, with a small fraction of PN tumor cells. Interestingly, we found that macrophages composed the larger fraction of non-tumor cells infiltrating the tumor core (P1/4/5/6/7) but they were replaced by microglia in the peritumoral invading front (P2/3). The peritumoral biopsies contained a higher fraction of non-tumor cells than glioma cells.

As scRNA-seq can be used to infer the underlying CNV, we interrogated our dataset to uncover the spatial dynamics of CNV changes in glioma cells to reconstruct the trajectory of tumor initiation and progression. Five CNV-based clusters (CN-1 to CN-5) were found in patient GS1 (Fig. 4B). CN-1 and CN-2 contained diploid non-tumor cells, but were divided into two clusters because their expression profiles differed from those of normal glial and immune cells. CN-3 to CN-5 clusters contained a series of aneuploid subclones that shared the *chr7*/*10*/*19*/*20* CNVs that are recurrent genomic alterations in GBM. As they progressed, these subclones first accumulated CNVs on *chr2* and later on *chr5* as they transitioned from CN-3 to CN-4 and from CN-4 to CN-5, respectively (Fig. 4C), therefore highlighting the temporal progression of this tumor. The combination of spatial and temporal information indicated that the CN-3 population was distributed only in P5/P6, whereas the CN-4 and CN-5 populations were present in all other tumoral and peritumoral sites (Fig. 4D). These findings clearly indicate that P5/P6 were the initial sites of the tumor that progressed towards the locations of P1/P4/P7 and P3 (Fig. 4A). In the global t-SNE analysis, as clone CN-3 developed into CN-4 and CN-5, glioma cells also transformed from cluster 9 to 20 (Fig. 4E). Our findings established that the number of somatic CNVs increased across the different glioma regions, thus defining a pattern of tumor progression. They are also consistent with the notion that an increased definition of the multi-sector sampling of glioma might reveal finer and more accurate genetic trajectories of glioma evolution, as shown in recent hepato-carcinoma studies [24].

To unravel the phenotypic changes that mark spatial evolution of glioma, we performed differential gene expression analysis between the different clones. As cells classified within the CN-3 clone were replaced by those in the CN-4 and CN-5 clones, we observed increased expression of genes implicated in negative regulation of cell differentiation (*HES1*, *HSPA9*, *ID2*), DNA damage response (*MDM4*, *SOX4*) and chemoattractant cytokine and neutrophil activation (*MAPK14*, *S100A11*), by general function annotation. The transition from CN-3 to CN-4 and CN-5 clones coincided also with increased expression of the *EGFR* oncogene. Conversely, genes expressed in benign astrocytes (*CNR1*, *CHRNA1*, *LAMA2*, *GNG11*, *GRIA2*, *GRIA3*, *GRIA4*) and general endothelial cell differentiation markers (*ARHGEF26*, *CLIC4*, *APOLD1*) were suppressed during the transition (Fig. 4F and G, Supplementary Table 2). Considering the functional enrichment of differentially expressed genes, our findings converge on a model whereby the genetic alterations such as CNVs that accumulate during turn or invasion lead to loss of differentiated astrocyte properties and gain of known features driving tumor aggressiveness, angiogenesis, dedifferentiation and oncogenic *PI3K*/*AKT* signaling (Fig. 4H), ultimately resulting in the promotion of glioma progression towards more aggressive and invasive phenotypes.

### Trajectory of tumor cell states reveals branched progression in patient GS13

**Figure 5.**
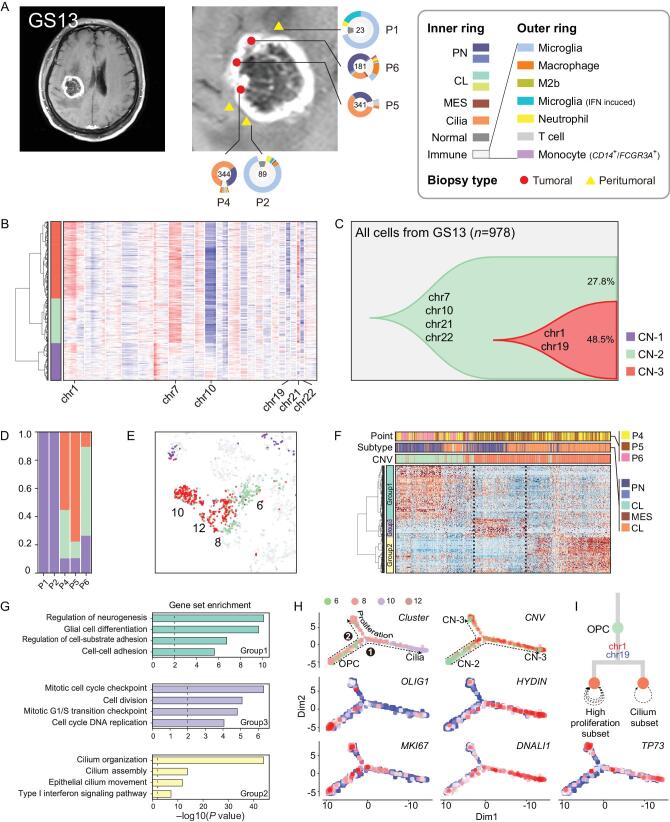
Glioma clonal evolution of Patient GS13 at spatial and temporal resolution. (A) MRI image of Patient GS13. Yellow and red markers in zoomed image represent peritumoral and tumoral sampling points. Ring plot in the right and bottom displays cell components of each point. Color of the inner ring shows classified glial cell subtypes, and the outer ring shows detailed immune cell subtypes. Cell numbers are labeled in the center of these ring plots. (B) Single-cell CNV heatmap, cells are divided into three groups by hierarchical clustering. (C) Clonal evolution trail followed by accumulating CNV events. Each color represents a CNV subclone and chromosomes are labeled in which copy number alterations occurred during clonal transition. (D) CNV subclone components in each sampling point, P6 had a lower CN-3 ratio than P4/5. (E) CNV subclone distribution in t-SNE coordinates. When CN-2 developed into CN-3, cells translocated from cluster 6 to cluster 12/10. (F) Heatmap of differentially expressed genes between CN-2/5 glial cells. Hierarchical clustering was applied in both gene and cell dimensions. Differential genes could be divided into three gene-sets, marked with the left color bar. (G) Functional enrichment of three gene-sets in (F). (H) Single-cell trajectories of malignant cells in GS13. Top left subplot colored by global t-SNE clusters, two branches of cells were developed from OPC cells. The top right subplot is colored by CNV group. The remaining four subplots were relative expression patterns of marker genes (*OLIG2*, *HYDIN*, *MKI67*, *DNALI1* and *TP73*). (I) Branched clonal developing model of GS13.

Patient GS13 was a male with an *IDH*-wild type GBM characterized by high expression of genes associated with motile cilium activities (e.g. *FOXJ1*, *FAM183A*, *HYDIN*, *DNALI1*, etc.) (Supplementary Figs S8 and S10). We noted that only about 5% of TCGA GBM patients exhibited high expression of cilium-related genes. Therefore, the particular type of GBM analyzed from patient GS13 belongs to a rare type of glioma that was not previously investigated at single-cell level. However, cilium-specific gene expression was not associated with a specific pattern of survival (Supplementary Fig. S11). From this patient, we acquired 978 cells from three core and two peritumoral sites, and found that the cells at core locations consisted of PN and cilium-positive cells (Fig. 5A). CNVs were also evident in this case. The clonal CNV reconstruction revealed two CNV clones (CN-2 and CN-3) and the transition from CN-2 to CN-3 was marked by accumulation of *chr1^amp^* and *chr19^del^*. Furthermore, as cells transitioned to CN-3, the expression profile changed from global cluster 6 (PN) to global clusters 10 and 12 (cilium) (Fig. 5B–E). We also applied hierarchical clustering of CN-2 and CN-3 malignant cells and identified differentially expressed genes between these two groups. Interestingly, after cells transitioned to CN-3, they formed two branches with distinct gene expression profiles (Fig. 5F and H, Supplementary Table 2). The functional gene-set enrichment analysis showed that the transition from CN-2 to CN-3 was associated with decreased expression of group 1 genes, which were implicated in glial cell differentiation and adhesion. Conversely, the expression of genes in groups 2 and 3 (implicated in cell cycle/DNA replication and cilium regulation, respectively) increased (Fig. 5G and Supplementary Fig. S12).

To define the pattern of progression of this branched trail, we analyzed the single-cell trajectory of these cells with Monocle2 [25]. In a pseudotime model, this trail started from global cluster 6, which overexpressed oligodendrocyte progenitor cell marker *OLIG1*. Then, the trail branched into two directions when CN-2 became CN-3, and branches 1 and 2 correspond to global clusters 10 and 12, respectively. Branch 1 exclusively expressed cilium markers (e.g. *HYDIN*, *FOXJ1*, *DNALI1*, etc.) (Fig. 5H and I), while branch 2 maintained the PN nature but showed high proliferative ability. Overexpression of *TP73* and *HYDIN* was validated by immunohistochemistry (IHC) staining experiments (Supplementary Fig. S13). Moreover, expression of the *TP73* gene at *chr1p36* increased in *chr1^amp^* cells (Fig. 5H). From previous studies, a protumorigenic activity of *TP73* has recently emerged, especially in the context of the N-terminal truncated *TP73* isoform [26]. Furthermore, *TP73* overexpression has been reported in several tumor types, including breast cancer, melanoma, prostate cancer and neuroblastoma, and was shown to induce metastasis, chemo-resistance and other hallmarks of tumor progression that confer poor clinical outcome [27]. By RT-qPCR detection, both full length and *ΔN-TP73* existed in GS13 tumor (Supplementary Fig. S14).

In conclusion, the expression of a motile cilium signature is not an unusual event in GBM, but little is known about this particular GBM phenotype. The branched model revealed that glioma cells may go through different spatial destinations despite sharing similar CNV profiles.

### Characterization of the TME in glioma

**Figure 6.**
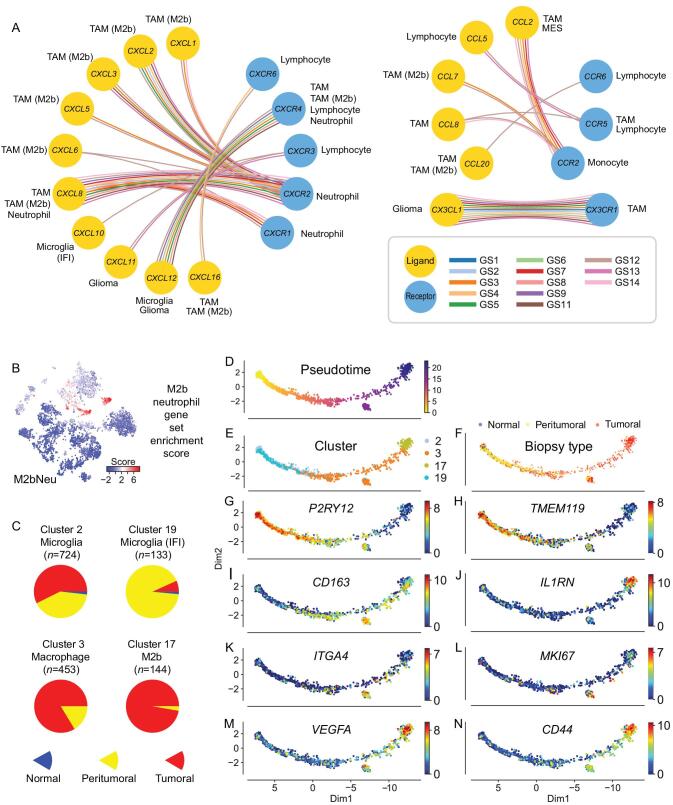
Characteristics of M2b macrophages and neutrophils and their potential in prognosis prediction using M2bNeu score. (A) Major chemokine and chemokine receptors relationship inside glioma tissue. (B) Dot plots show the M2bNeu score distribution in the optimized global t-SNE map. These scores were calculated by M2bNeu genes listed in Supplementary Fig. 14C. (C) Biopsy type distribution of tumor associated microglia and macrophage cells. (D) Trajectory analysis of macrophage/microglia evolution on TAM cells from all patients, colored by (D) pseudotime, (E) optimized global t-SNE clusters, (F) biopsy types and (G–N) marker genes expressed in the pseudotime trajectory map.

In the glioma TME, tumor-associated macrophages (TAM) communicate through ligand/receptor cross-talks with tumor and non-tumor cells to promote tumor aggressiveness [28,29]. As our single-cell platform exhibits high sensitivity with a higher number of average detectable genes expressed in single cells compared to other glioma datasets (4470 genes/cell versus <2000 genes/cell), we sought to build a ligand/receptor interaction map for reconstruction of the most important chemoattractant relationships that exist between glioma tumor cells and TAM in the glioma TME (Fig. 6A and Supplementary Fig. S15). Overall, we detected 16 chemokine ligands and nine receptors in 13 patients.

Microglia and M2a/c macrophages, which expressed the *CX3CR1* receptor, coexisted with glioma cells that expressed the *CX3CL1* ligand. Lymphocyte infiltrates expressed *CXCR3*/*6* and *CCR6*, but the *CXCR3* ligands *CXCL9*/*10*/*11* were rarely detected in glioma samples with the exception of interferon-activated microglia. These ligand–receptor pairs were previously reported to recruit tumor-infiltrating lymphocytes and inhibit tumor growth [30]. *CXCR6* ligand *CXCL16*, which exists in both transmembrane and soluble form [31], was highly expressed in TAM cells, and mildly expressed in the malignant cells. Lymphocytes also expressed *CXCR4* like TAM cells, whereas the ligands were expressed in microglia cells. Their binding was reported to mediate glioma chemotaxis and regulate cell survival through activating *AKT*-related pathways [32].

*CCL5*/*CCL8* and *CCR5* was another chemokine axis between lymphocytes and TAM. Besides inflammatory chemoattractant functions, this can also mediate NK cell activation, which promotes tumor genesis and metastasis [33]. Another receptor of *CCL8* is *CCR2*, which is expressed in monocytes, and highly expressed in TAM cells and MES cells.

A strong impact from the *CXCL* family and related receptors was found in M2b macrophages and neutrophils (Supplementary Fig. S16A and B). *CXCL1*/*2*/*3*/*5*/*6*/*8* were overexpressed in M2b cells, generating a chemoattractant environment that might recruit *CXCR1^+^*/*CXCR2^+^* neutrophils. In glioma, M2b polarization and recruiting neutrophils have been connected with pro-tumoral functions [34–36]. Based on their common gene expression signals, we calculated an enrichment score from 38 genes (Fig. 6B and Supplementary Fig. S16C) and used this signature to deconvolute the presence of these cells from gene expression profiles of bulk tissues and predict clinical outcome. This method had been validated with TCGA glioma RNA-seq datasets (Supplementary Fig. S16D and E).

In our spatial cell distribution data, macrophages were the most abundant non-tumor cell in core biopsies, but were replaced by larger fractions of microglial cells in biopsies from the tumor margins (Fig. 6C). Thus, a switch from macrophage to microglia infiltration from the glioma core to the periphery was a general event that likely marked the microenvironmental changes. The microenvironment changes are likely to be dictated by different requirements of glioma cells as they migrate from core tumor regions to the invading front at the tumor periphery. This pattern was also recapitulated in the IVY GAP dataset [37] (Supplementary Fig. S17).

Finally, a lineage trajectory was built with TAM cells (Fig. 6D–F), showing the gradual change of three transitional states. The pattern of TAM development in glioma started first with a microglia phenotype (*P2RY12*^+^/*TMEM119*^+^), then it turned to polarized macrophage (*CD163*^+^), and finally converged into M2b macrophages (*IL1RN*^+^) with activated expression of strong angiogenesis signaling molecules (*VEGFA*). In the middle of this trajectory, we detected a small branch of cells that expressed high levels of the bone-marrow-derived macrophage (BMDM) TAM marker *ITGA4*. These findings suggest a model whereby TAM cell polarization in glioma is the result of two independent cell sources: resident microglial cells and BMDM cells (Fig. 6G–N). As we have also been able to compare the cell fates of TAM between the different grades of glioma, we found that they exhibited lower M2 polarization in LGG samples (GS8/9/14) when compared to GBM (Supplementary Fig. S18).

## DISCUSSION

As malignant gliomas are characterized by high degree of intratumoral heterogeneity, single-cell genomic technologies have rapidly emerged as a crucial approach to disentangle glioma heterogeneity. However, most of the previous single-cell RNA-seq glioma studies relied on the analysis of a single biopsy from each tumor specimen, thus lacking information on the special heterogeneity of the analyzed tumor lesions [6–9]. Although one study reported the multi-regional analysis of glioma with single-cell sequencing, the number of cells analyzed in that study was very limited and could not provide a comprehensive picture of the geographical structure of glioma at the single-cell level [10].

Here, we presented a comprehensive single-cell landscape of multiple subtypes of gliomas, each of which was analyzed by multi-region samplings, and provided the first spatial-level analysis of the cellular states that characterize human gliomas. We designed multi-sector biopsies with a 3D-enhanced MRI model, and collected them during surgery by navigation sampling. For each biopsy, we generated and functionally annotated transcriptomes of hundreds of single tumor and non-tumor cells collected from multiple core and periphery tumor locations. Together, they provide a coherent map of the dynamic states and interactions between the different cell types that integrate the key features of glioma homeostasis at each tumor location. We found that both the number and the transcriptomic subtypes assigned to individual glioma cells frequently change dramatically between biopsies collected from different locations, even when they originated from neighboring glioma regions. We also made the unexpected observation that whereas core biopsies contained a high number of macrophages, this configuration of the core TME was replaced by a comparatively higher number of resident microglia at the glioma periphery, which represents the invading front of the tumor towards the normal brain.

As malignant glioma cells share high proliferation capacity, they readily accumulate multiple types of genetic alterations that trigger an increasing degree of aneuploidy with constant adaptation to the demands created by the growing tumor mass in relation to the TME [38]. A glioma cell CNV-driven progression trajectory we uncovered that was especially highlighted by the dynamic changes in the tumor from patient GS1 was marked by progressive loss of the astrocyte-like hallmarks of glioma cells with gain of multiple tumor cell phenotypes (loss of differentiation, competence to migrate and invade through the extracellular matrix, etc.), which together drive glioma progression and invasion of the normal brain. Another progression trajectory we uncovered was well represented in the tumor of patient GS13, in which the pseudotime trajectory produced by Monocle2 identified a drastic switch in the major tumor cell population with gain of an intriguing ciliated phenotype that likely contributes to glioma aggressiveness. These transformation models indicate that the constant rearrangement of the genome of glioma tumor cells leads to continuous gain of new capacities, all of which converge towards the acquisition of more aggressive tumor phenotypes for always more deregulated proliferation, anaplasia and invasion of the normal brain.

An important, novel finding contributed by our work is the deconvolution of the cross-talks between tumor and non-tumor cells in the glioma TME. We found that the active communications between the different cell types are primarily implemented by multiple combinations of chemokine ligands with their corresponding receptors that we have characterized within different regions of individual tumors and among the different types of glioma we studied [39]. In particular, we found that the communication between non-tumor cells was dominated by the prominent role of the *CXCL* family of chemokines and related receptors, which was especially apparent in M2b macrophages and neutrophils. We followed up on this finding and determined the enrichment score of M2b/neutrophil cells in bulk gliomas to evaluate the consequences of the infiltration of these cell types for clinical outcome of glioma patients. The analysis, which was performed with TCGA-derived glioma RNA-seq datasets, was able to distinguish patients with divergent clinical outcome based on the predicted level of infiltration of the two cell types.

In conclusion, we used the single-cell RNA-seq technology to generate an extensive complete map of the geographical molecular structure of gliomas. The trajectories of reciprocal genomic and functional changes that accompany glioma cells as they move within the tridimensional space of the tumor mass, combined with the deconvolution of the cross-talks between different cells in the glioma TME, paint an unprecedented scenario that elucidates the intratumoral heterogeneity of this lethal tumor type.

## METHODS AND MATERIALS

The detailed descriptions of methods are available as Supplementary Materials at *NSR* online.

## Supplementary Material

nwaa099_Supplemental_Files

## Data Availability

Both the single-cell RNA-seq data and bulk DNA-seq data we used in this study were submitted to Genome Sequence Archive (GSA) in the Beijing Institute of Genomics (BIG), Chinese Academy of Sciences, under accession number HRA000179 that are publicly accessible at https://bigd.big.ac.cn/gsa-human. Barcode sequences and prepared gene expression count matrix were also submitted to the Gene Expression Omnibus (GEO) under accession number GSE117891. Other information related to this study is available from the corresponding authors upon reasonable request.

## References

[1] Vogelstein B, Papadopoulos N, Velculescu VE et al. Cancer genome landscapes. Science 2013; 339: 1546–58. 23539594 10.1126/science.1235122 PMC3749880

[2] Snuderl M, Fazlollahi L, Le LP et al. Mosaic amplification of multiple receptor tyrosine kinase genes in glioblastoma. Cancer Cell 2011; 20: 810–7. 22137795 10.1016/j.ccr.2011.11.005

[3] Meyer M, Reimand J, Lan X et al. Single cell-derived clonal analysis of human glioblastoma links functional and genomic heterogeneity. Proc Natl Acad Sci USA 2015; 112: 851–6. 25561528 10.1073/pnas.1320611111 PMC4311802

[4] Sottoriva A, Spiteri I, Piccirillo SGM et al. Intratumor heterogeneity in human glioblastoma reflects cancer evolutionary dynamics. Proc Natl Acad Sci USA 2013; 110: 4009–14. 23412337 10.1073/pnas.1219747110 PMC3593922

[5] Suzuki H, Aoki K, Chiba K et al. Mutational landscape and clonal architecture in grade II and III gliomas. Nat Genet 2015; 47: 458–68. 25848751 10.1038/ng.3273

[6] Patel AP, Tirosh I, Trombetta JJ et al. Single-cell RNA-seq highlights intratumoral heterogeneity in primary glioblastoma. Science 2014; 344: 1396–401. 24925914 10.1126/science.1254257 PMC4123637

[7] Tirosh I, Venteicher AS, Hebert C et al. Single-cell RNA-seq supports a developmental hierarchy in human oligodendroglioma. Nature 2016; 539: 309–13. 27806376 10.1038/nature20123 PMC5465819

[8] Venteicher AS, Tirosh I, Hebert C et al. Decoupling genetics, lineages, and microenvironment in IDH-mutant gliomas by single-cell RNA-seq. Science 2017; 355: 1391. 10.1126/science.aai8478 PMC5519096 28360267

[9] Darmanis S, Sloan SA, Croote D et al. Single-Cell RNA-Seq analysis of infiltrating neoplastic cells at the migrating front of human glioblastoma. Cell Rep 2017; 21: 1399–410. 29091775 10.1016/j.celrep.2017.10.030 PMC5810554

[10] Lee J-K, Wang J, Sa JK et al. Spatiotemporal genomic architecture informs precision oncology in glioblastoma. Nat Genet 2017; 49: 594–9. 28263318 10.1038/ng.3806 PMC5627771

[11] Neftel C, Laffy J, Filbin MG et al. An integrative model of cellular states, plasticity, and genetics for glioblastoma. Cell 2019; 178: 835–49. 31327527 10.1016/j.cell.2019.06.024 PMC6703186

[12] Dong J, Hu Y, Fan X et al. Single-cell RNA-seq analysis unveils a prevalent epithelial/mesenchymal hybrid state during mouse organogenesis. Genome Biol 2018; 19: 31. 29540203 10.1186/s13059-018-1416-2 PMC5853091

[13] Gao S, Yan L, Wang R et al. Tracing the temporal-spatial transcriptome landscapes of the human fetal digestive tract using single-cell RNA-sequencing. Nat Cell Biol 2018; 20: 721–34. 29802404 10.1038/s41556-018-0105-4

[14] Aibar S, González-Blas CB, Moerman T et al. SCENIC: single-cell regulatory network inference and clustering. Nat Methods 2017; 14: 1083–6. 28991892 10.1038/nmeth.4463 PMC5937676

[15] Peschl P, Bradl M, Höftberger R et al. Myelin oligodendrocyte glycoprotein: deciphering a target in inflammatory demyelinating diseases. Front Immunol 2017; 8: 529. 28533781 10.3389/fimmu.2017.00529 PMC5420591

[16] Zhan C, Yan L, Wang L et al. Identification of immunohistochemical markers for distinguishing lung adenocarcinoma from squamous cell carcinoma. J Thorac Dis 2015; 7: 1398–405. 26380766 10.3978/j.issn.2072-1439.2015.07.25 PMC4561256

[17] Mei F, Wang H, Liu S et al. Stage-specific deletion of Olig2 conveys opposing functions on differentiation and maturation of oligodendrocytes. J Neurosci 2013; 33: 8454–62. 23658182 10.1523/JNEUROSCI.2453-12.2013 PMC3865513

[18] Saadoun S, Papadopoulos MC. Aquaporin-4 in brain and spinal cord oedema. Neuroscience 2010; 168: 1036–46. 19682555 10.1016/j.neuroscience.2009.08.019

[19] Verhaak RGW, Hoadley KA, Purdom E et al. Integrated genomic analysis identifies clinically relevant subtypes of glioblastoma characterized by abnormalities in PDGFRA, IDH1, EGFR, and NF1. Cancer Cell 2010; 17: 98–110. 20129251 10.1016/j.ccr.2009.12.020 PMC2818769

[20] Satir P, Christensen ST. Structure and function of mammalian cilia. Histochem Cell Biol 2008; 129: 687–93. 18365235 10.1007/s00418-008-0416-9 PMC2386530

[21] Yu X, Ng CP, Habacher H et al. Foxj1 transcription factors are master regulators of the motile ciliogenic program. Nat Genet 2008; 40: 1445–53. 19011630 10.1038/ng.263

[22] Casasent AK, Schalck A, Gao R et al. Multiclonal invasion in breast tumors identified by topographic single cell sequencing. Cell 2018; 172: 205–17. 29307488 10.1016/j.cell.2017.12.007 PMC5766405

[23] Wang L, Fan J, Francis JM et al. Integrated single-cell genetic and transcriptional analysis suggests novel drivers of chronic lymphocytic leukemia. Genome Res 2017; 27: 1300–11. 28679620 10.1101/gr.217331.116 PMC5538547

[24] Ling S, Hu Z, Yang Z et al. Extremely high genetic diversity in a single tumor points to prevalence of non-Darwinian cell evolution. Proc Natl Acad Sci USA 2015; 112: 6496–505. 10.1073/pnas.1519556112 PMC4664355 26561581

[25] Trapnell C, Cacchiarelli D, Grimsby J et al. The dynamics and regulators of cell fate decisions are revealed by pseudotemporal ordering of single cells. Nat Biotechnol 2014; 32: 381–6. 24658644 10.1038/nbt.2859 PMC4122333

[26] Stiewe T, Tuve S, Peter M et al. Quantitative TP73 transcript analysis in hepatocellular carcinomas. Clin Cancer Res 2004; 10: 626–33. 14760085 10.1158/1078-0432.ccr-0153-03

[27] Buhlmann S, Pützer BM. DNp73 a matter of cancer: mechanisms and clinical implications. Biochim Biophys Acta 2008; 1785: 207–16. 18302944 10.1016/j.bbcan.2008.01.002

[28] Quail DF, Joyce JA. The Microenvironmental landscape of brain tumors. Cancer Cell 2017; 31: 326–41. 28292436 10.1016/j.ccell.2017.02.009 PMC5424263

[29] Cohen M, Giladi A, Gorki A-D et al. Lung single-cell signaling interaction map reveals basophil role in macrophage imprinting. Cell 2018; 175: 1031–44. 30318149 10.1016/j.cell.2018.09.009

[30] Groom JR, Luster AD. CXCR3 in T cell function. Exp Cell Res 2011; 317: 620–31. 21376175 10.1016/j.yexcr.2010.12.017 PMC3065205

[31] Deng L, Chen N, Li Y et al. CXCR6/CXCL16 functions as a regulator in metastasis and progression of cancer. Biochim Biophys Acta 2010; 1806: 42–9. 20122997 10.1016/j.bbcan.2010.01.004

[32] Zhou Y, Larsen PH, Hao C et al. CXCR4 is a major chemokine receptor on glioma cells and mediates their survival. J Biol Chem 2002; 277: 49481–7. 12388552 10.1074/jbc.M206222200

[33] Arango Duque G, Descoteaux A. Macrophage cytokines: involvement in immunity and infectious diseases. Front Immunol 2014; 5: 491. 25339958 10.3389/fimmu.2014.00491 PMC4188125

[34] Balkwill FR . The chemokine system and cancer. J Pathol 2012; 226: 148–57. 21989643 10.1002/path.3029

[35] Qian B-Z, Pollard JW. Macrophage diversity enhances tumor progression and metastasis. Cell 2010; 141: 39–51. 20371344 10.1016/j.cell.2010.03.014 PMC4994190

[36] Turner MD, Nedjai B, Hurst T et al. Cytokines and chemokines: at the crossroads of cell signalling and inflammatory disease. Biochim Biophys Acta 2014; 1843: 2563–82. 24892271 10.1016/j.bbamcr.2014.05.014

[37] Puchalski RB, Shah N, Miller J et al. An anatomic transcriptional atlas of human glioblastoma. Science 2018; 360: 660–3. 29748285 10.1126/science.aaf2666 PMC6414061

[38] Wu C-I, Wang H-Y, Ling S et al. The ecology and evolution of cancer: the ultra-microevolutionary process. Annu Rev Genet 2016; 50: 347–69. 27686281 10.1146/annurev-genet-112414-054842

[39] Mollica Poeta V, Massara M, Capucetti A et al. Chemokines and chemokine receptors: new targets for cancer immunotherapy. Front Immunol 2019; 10: 379. 30894861 10.3389/fimmu.2019.00379 PMC6414456

