# Supplementary material for: Surveying brain tumor heterogeneity by single-cell RNA-sequencing of multi-sector biopsies
Source: Natl Sci Rev. 2020 May 30;7(8):1306–18. doi: 10.1093/nsr/nwaa099 (PMC8289159; doi:10.1093/nsr/nwaa099)
Supplement: nwaa099_Supplemental_Files [file nwaa099_supplemental_files.zip › nwaa099_Supplemental_File.docx]

# Supplementary Information

# Supplementary Figures

**
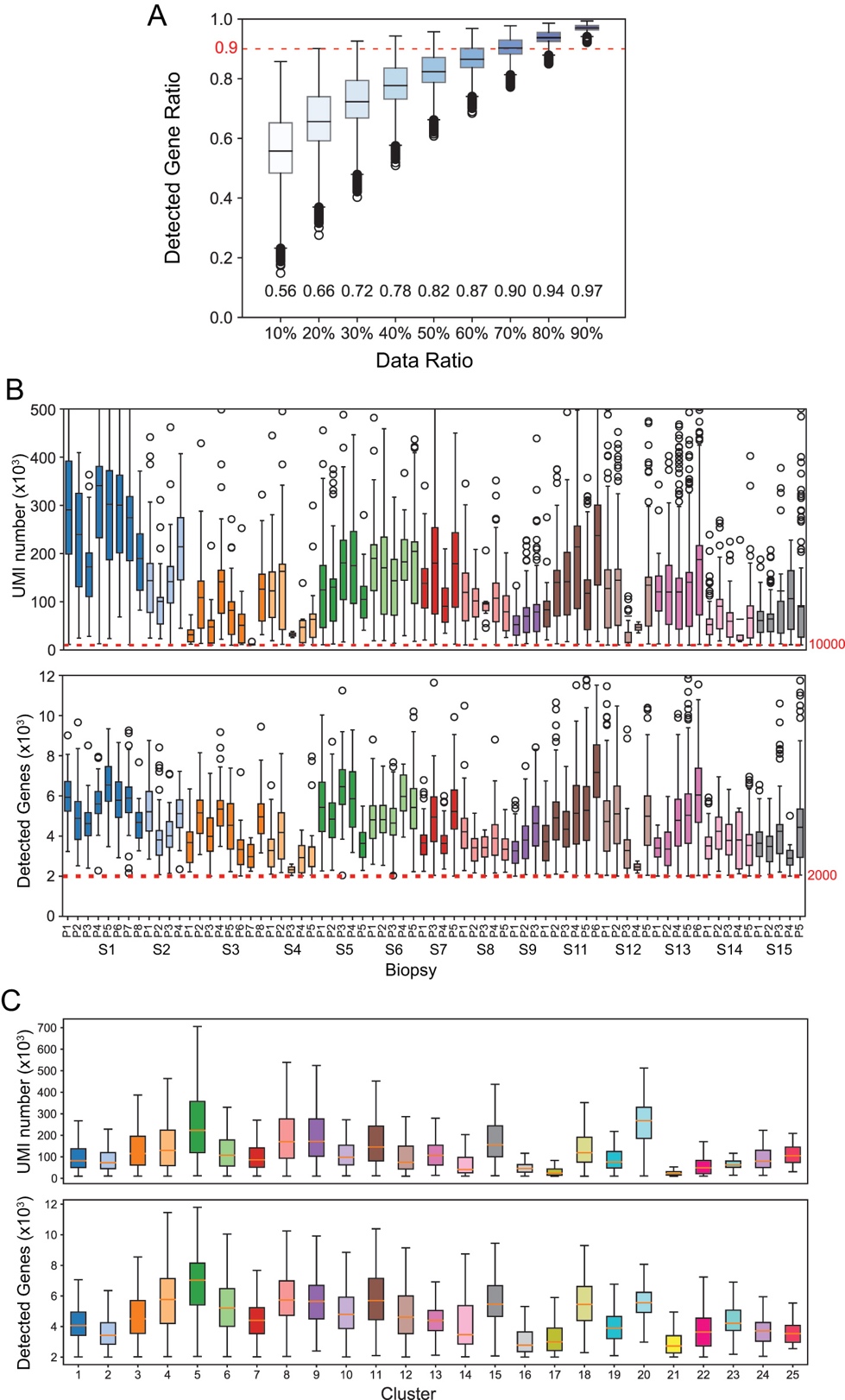
**

**Supplementary Fig. S1.** General Information of Experiment Procedure and Generated Data.

(A) Saturation analysis. Down sampling were done by reduce raw sequencing data to 10, 20, 30, 40, 50, 60, 70, 80 and 90% of its original data, thus calculated a series of ratio that describe detected genes proportion compared to using all raw data. (B) Boxplot of data summary, grouped by tumor biopsies, described UMI number and detected gene number of all quality filtered cells. (C) Boxplot of data summary, grouped by optimized global t-SNE clusters, described UMI number and detected gene number of all quality filtered cells.

**
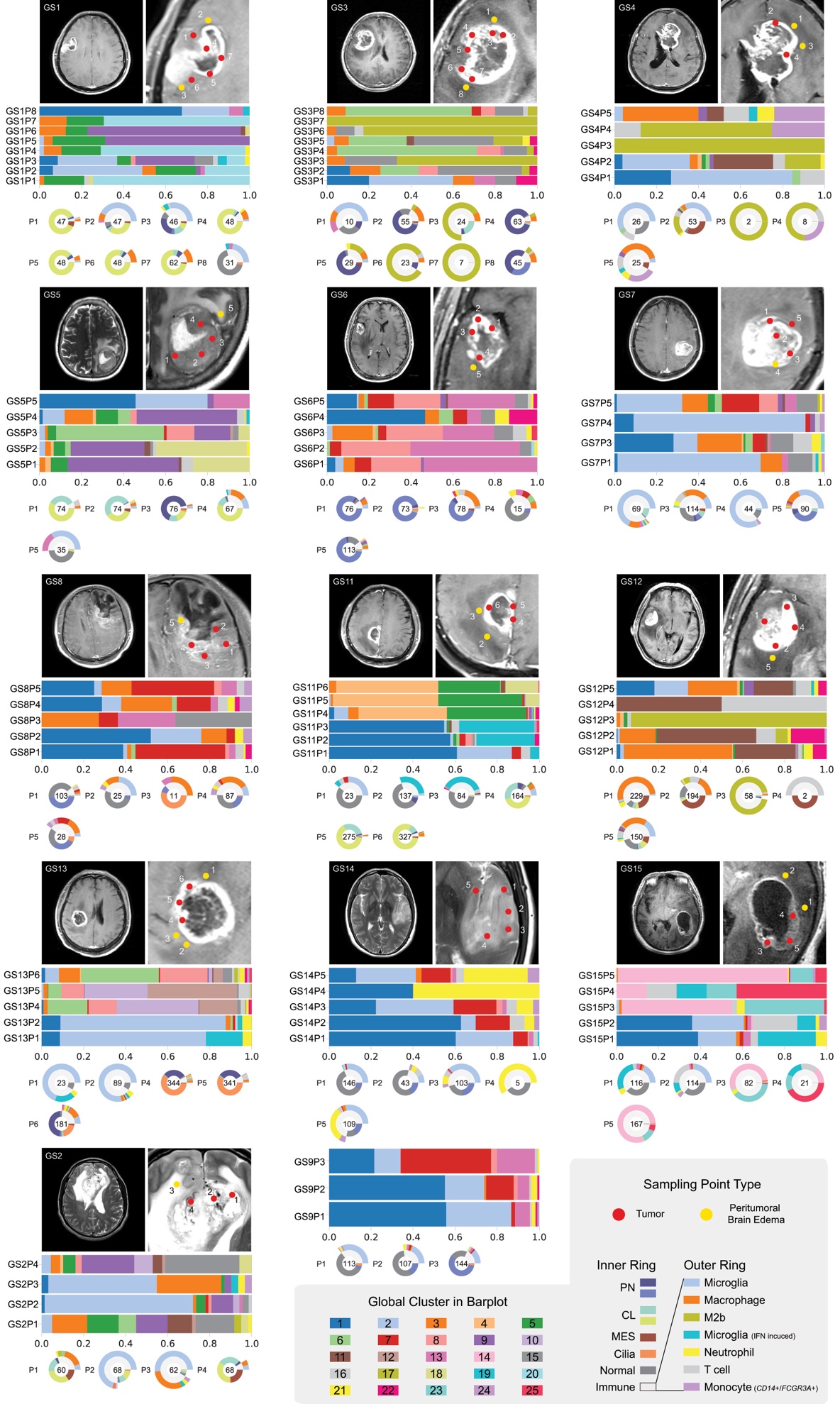
**

**Supplementary Fig. S2.** Medical Imaging and Cell Components of All Sampling Points in Each Patient.

Red and yellow dots marked locations of tumoral and peritumoral sampling point in the MRI image. Most of these MRI images were gadolinium enhanced except GS2. Bar plots illustrated cell components of each sampling points, representing 25 clusters in global t-SNE map, in accordance with the color code in (Fig. 1A and C). Ring plot illustrated cell components referred to TCGA 4 subtype classification (inner ring) and immune related cell types (outer ring). Cell number of each biopsy marked in the center of rings.

**Supplementary Fig. S3.** CNV Information at Bulk and Single-Cell Level.

(A) CNV subtype distributions in t-SNE map. (B) Bulk CNV status of sampling points by low depth whole genome sequencing. (C) Heatmaps demonstrated RNA-seq derived CNV pattern at single-cell level, each row is a single-cell, and hierarchical clustering divided these cells into several groups. Corresponding distributions were mapped in the global t-SNE coordinates.

**
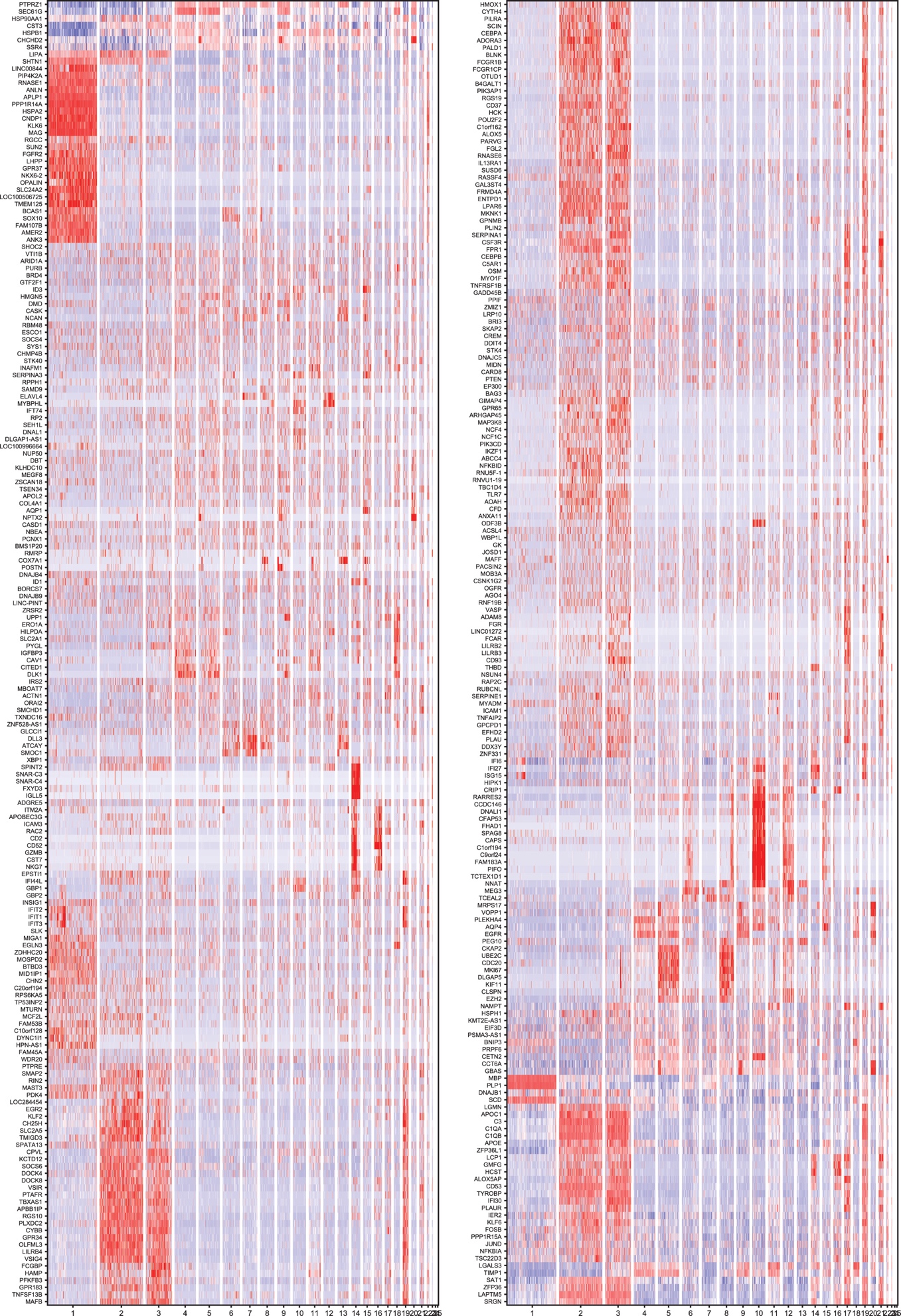
**

**Supplementary Fig. S4.** Gene expression heatmap of genes removed in the PCA and t-SNE analysis. These genes are located on chromosome regions that containing major CNV events.

**
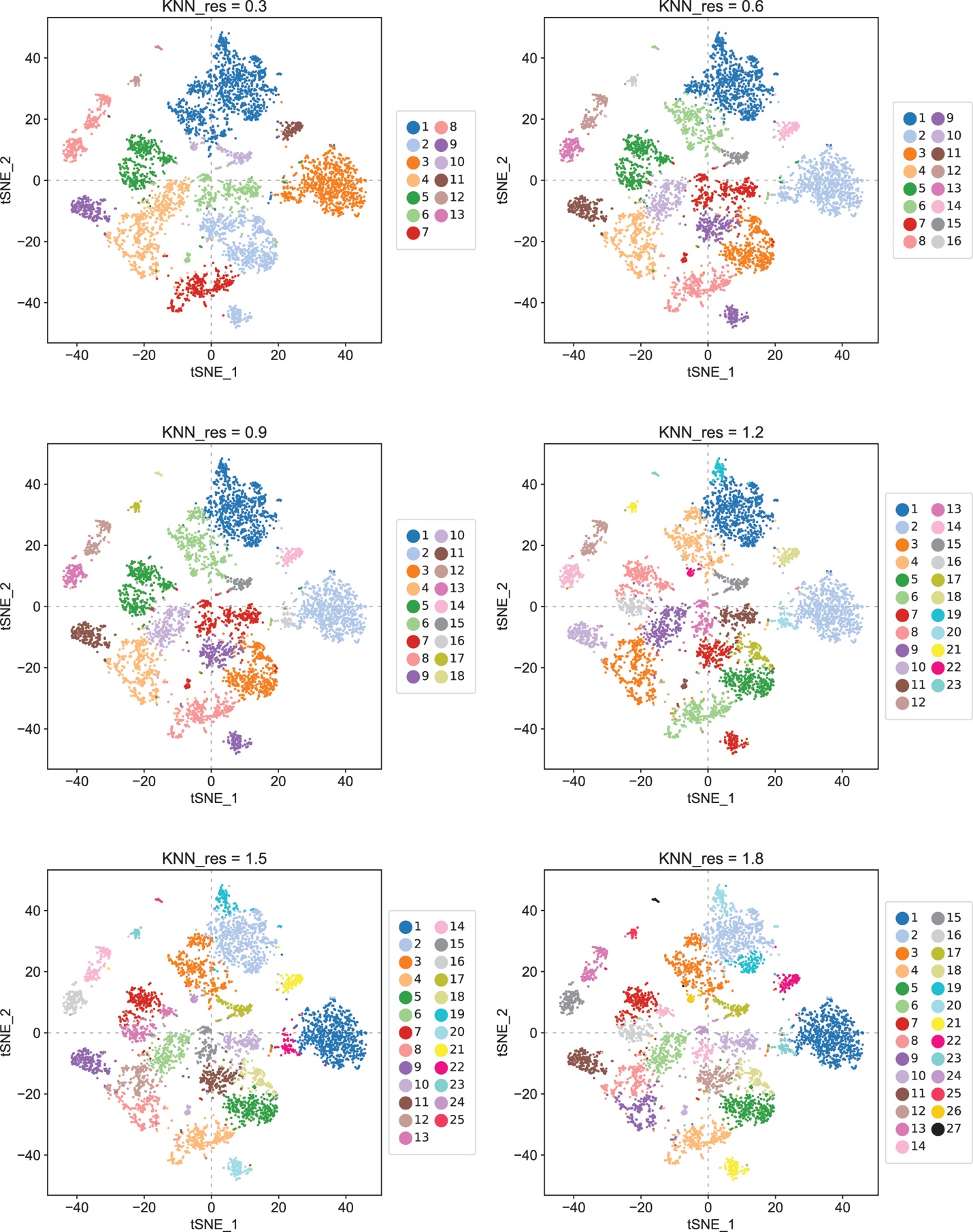
**

**Supplementary Fig. S5.** KNN clusters obtained with variable values of the resolution parameter.

**
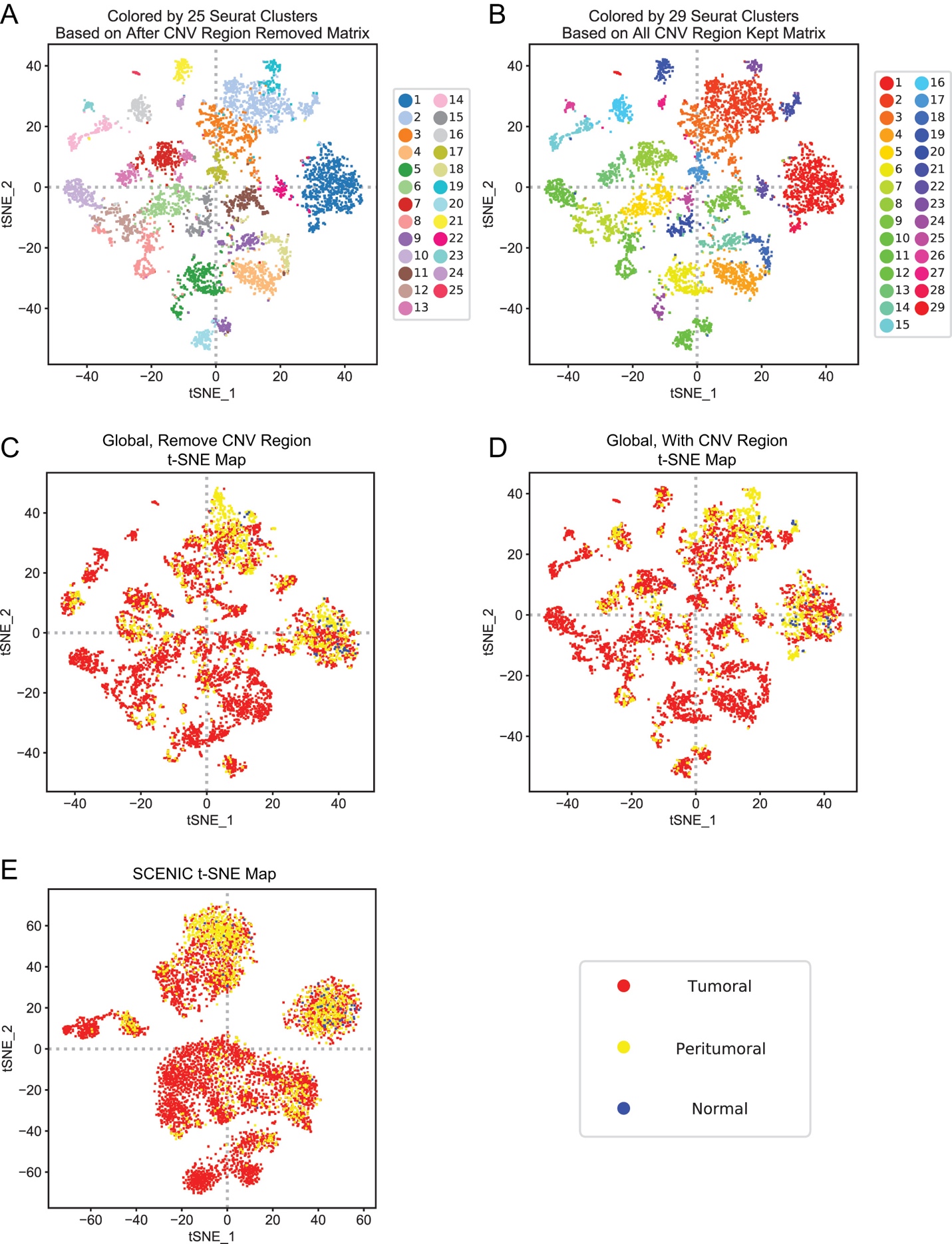
**

**Supplementary Fig. S6.** Comprehensive single cell information in different dimension reduction coordinates.

(A-B), Coordinates based on all variable genes Seurat t-SNE analysis, colored by (A) optimized global t-SNE clusters and (B) all variable genes derived original global t-SNE clusters. (C-F) Cells colored by biopsy types, mapped on (C) optimized Seurat t-SNE coordinates, (D) all variable genes derived original Seurat t-SNE coordinates, (E) SCENIC regulon based t-SNE.

**
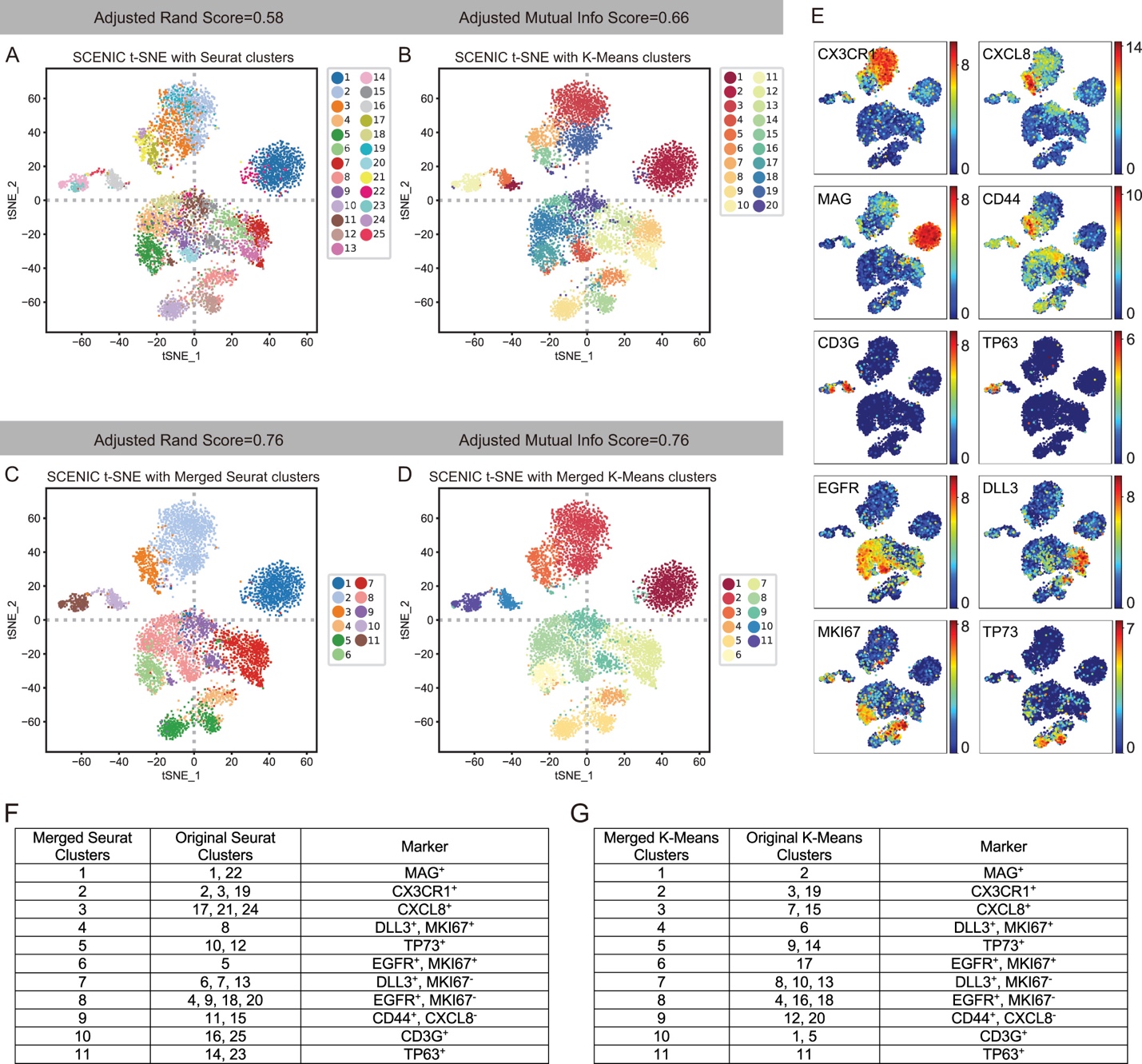
**

**Supplementary Fig. S7.** Comparative analysis of dimension reduction analysis by Seurat based regular gene expression matrix and SCENIC based regulon matrix.

(A) SCENIC t-SNE colored with Seurat clusters; (B) K-Means clusters; (C) Merged Seurat clusters and (D) Merged K-Means clusters. Adjusted rand score and adjusted mutual info score are calculated and listed in two groups: (A) and (B), (C) and (D). (F-G) Table of merged Seurat and regulon K-Means clusters, based on the marker gene expression pattern showed in (E).

**
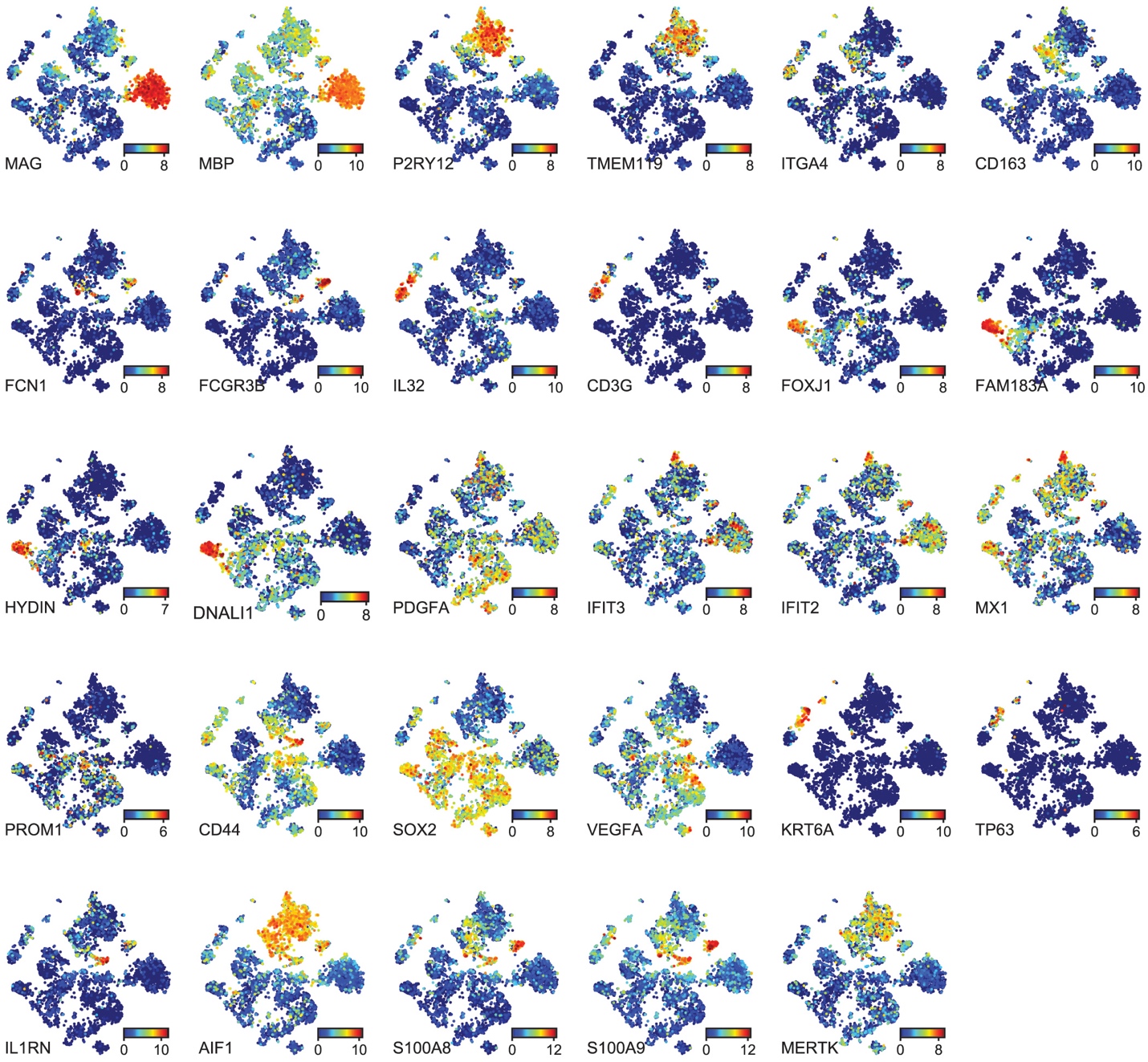
**

**Supplementary Fig. S8.** Marker gene expression feature map for cell type identification.

**
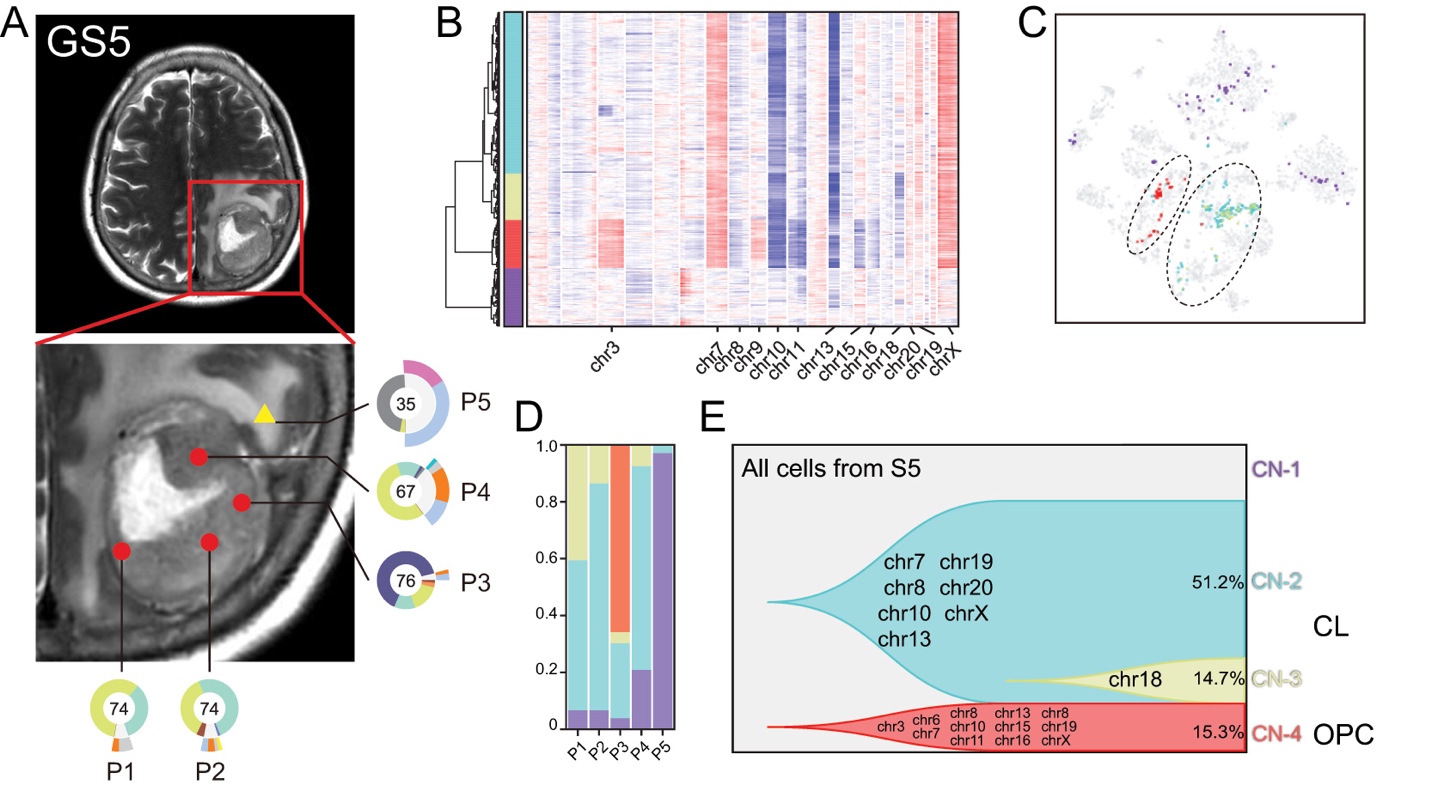
**

**Supplementary Fig. S9.** Glioma clonal evolution of Patient GS5 at spatial and temporal resolution. (A) MRI image of Patient GS5. Yellow and red markers in zoomed image represent peritumoral and tumoral sampling points. Ring plot in the right and bottom displayed cell component of each point. Color of the inner ring showed classified glial cell subtypes, and the outer ring showed detailed immune cell subtypes. Cell numbers were labeled in the center of these ring plots. (B) Single-cell CNV heatmap. Cells were divided into 4 groups by hierarchical clustering. (C) CNV subclone distributions in t-SNE coordinates. All of the CN-2 and CN-3 group are classical subtype, CN-4 cells demonstrated significant OPC properties. (D) CNV subclone components in each sampling point. OPC cells exclusively exist and form the major component of biopsy P3. Later stage classical CN-3 cells showed an increasing gradient in biopsies by the order of P3, P4, P2 and P1. (E) Clonal evolution trail followed by accumulating CNV events. Each color represented a CNV subclone and chromosomes were labeled, in which copy number alteration were happened during clonal transition. CN-3 have accumulated chr18del during tumorigenesis.

**
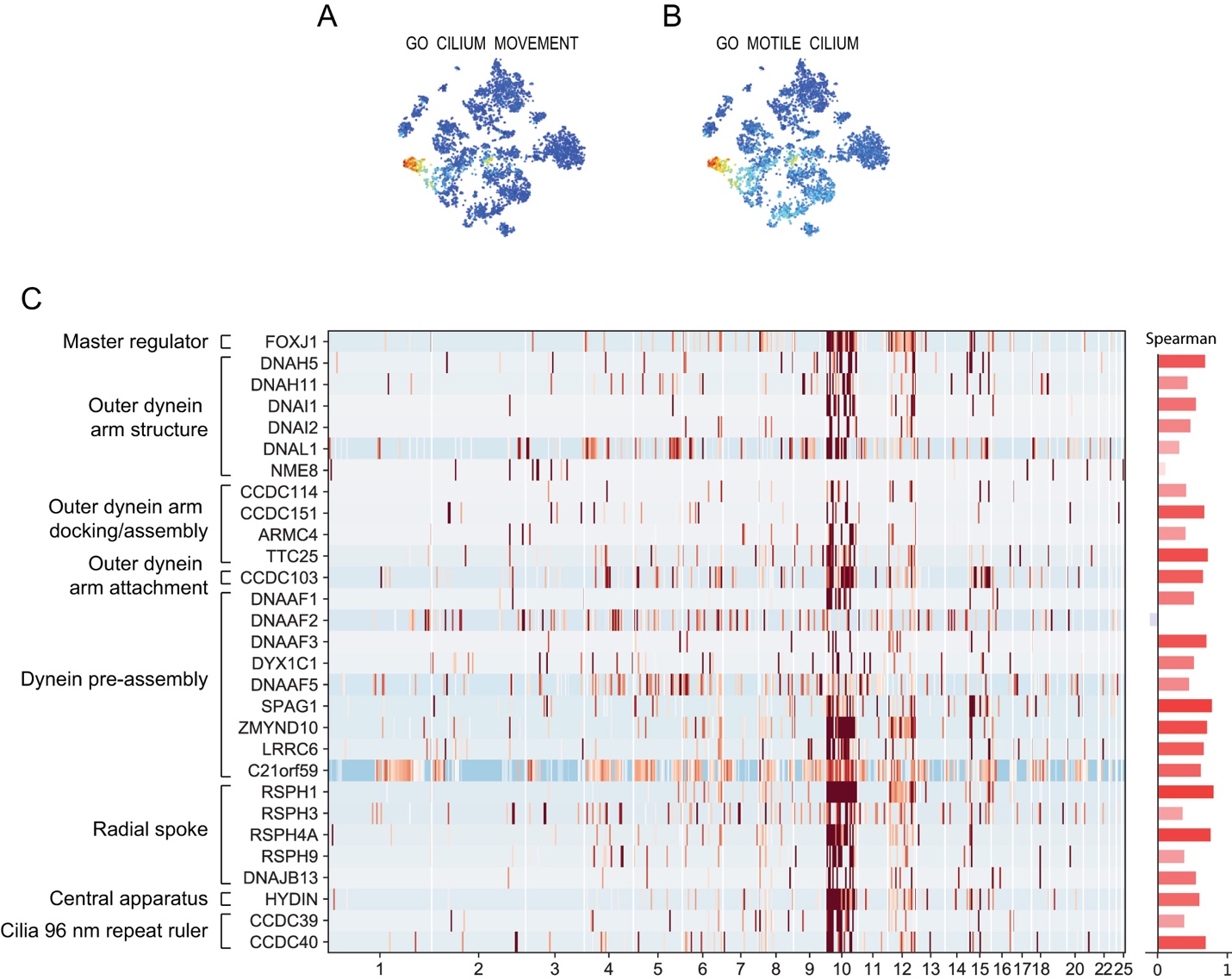
**

**Supplementary Fig. S10.** GS13 cilium cells overexpress motile cilium related marker genes. (A-B) Gene set score of motile cilium related GO terms. (C) Left panel: Expression heatmap of motile cilium related marker genes. Right panel: Spearman correlation coefficient values of marker genes to the master regulator *FOXJ1*.

**
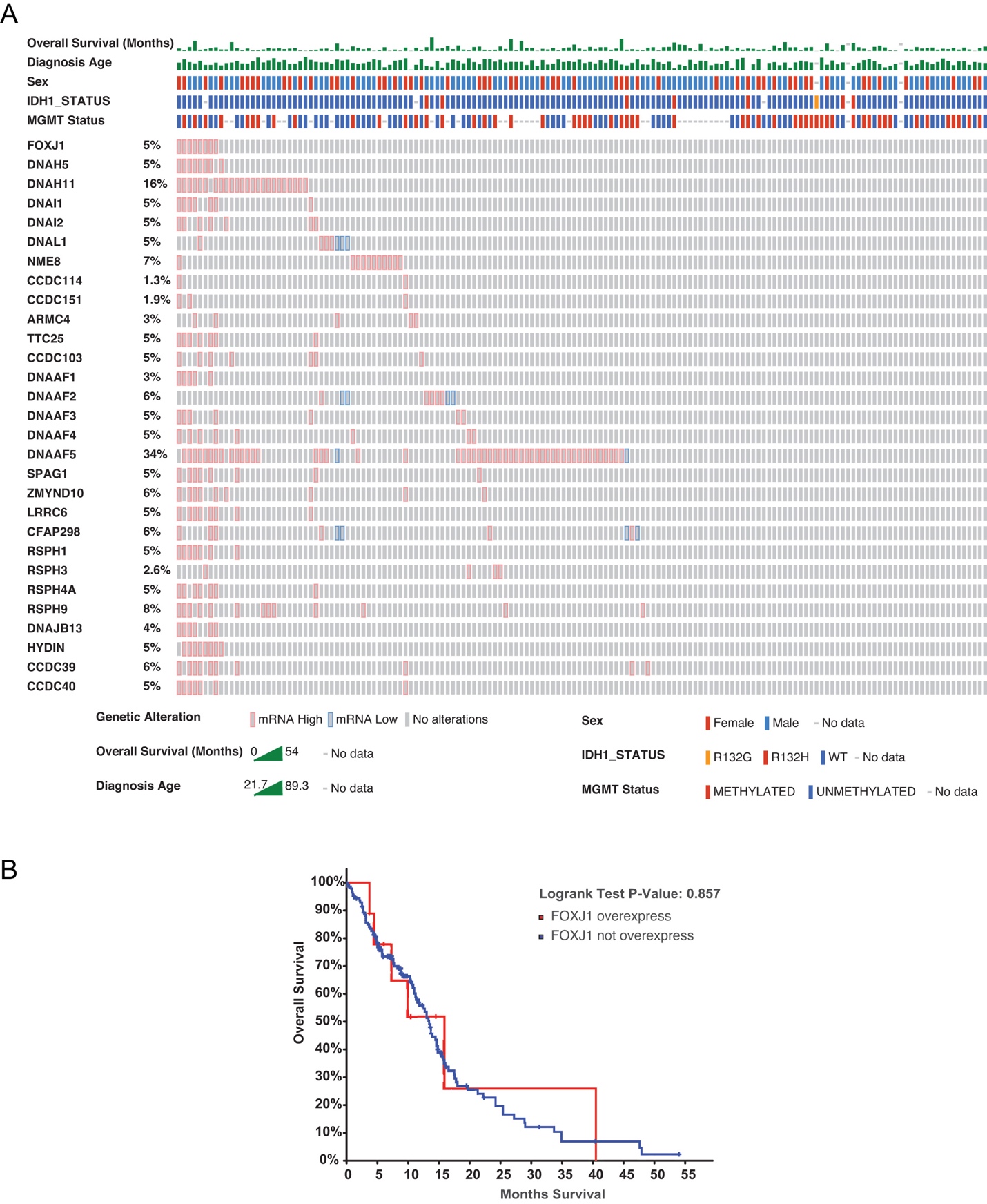
**

**Supplementary Fig. S11.** Motile cilium marker overexpression samples in TCGA GBM cohort. (A) Overexpressed motile cilium related genes in TCGA GBM cohort. (B) Overall survival analysis of *FOXJ1* overexpressed TCGA GBM patients.

**
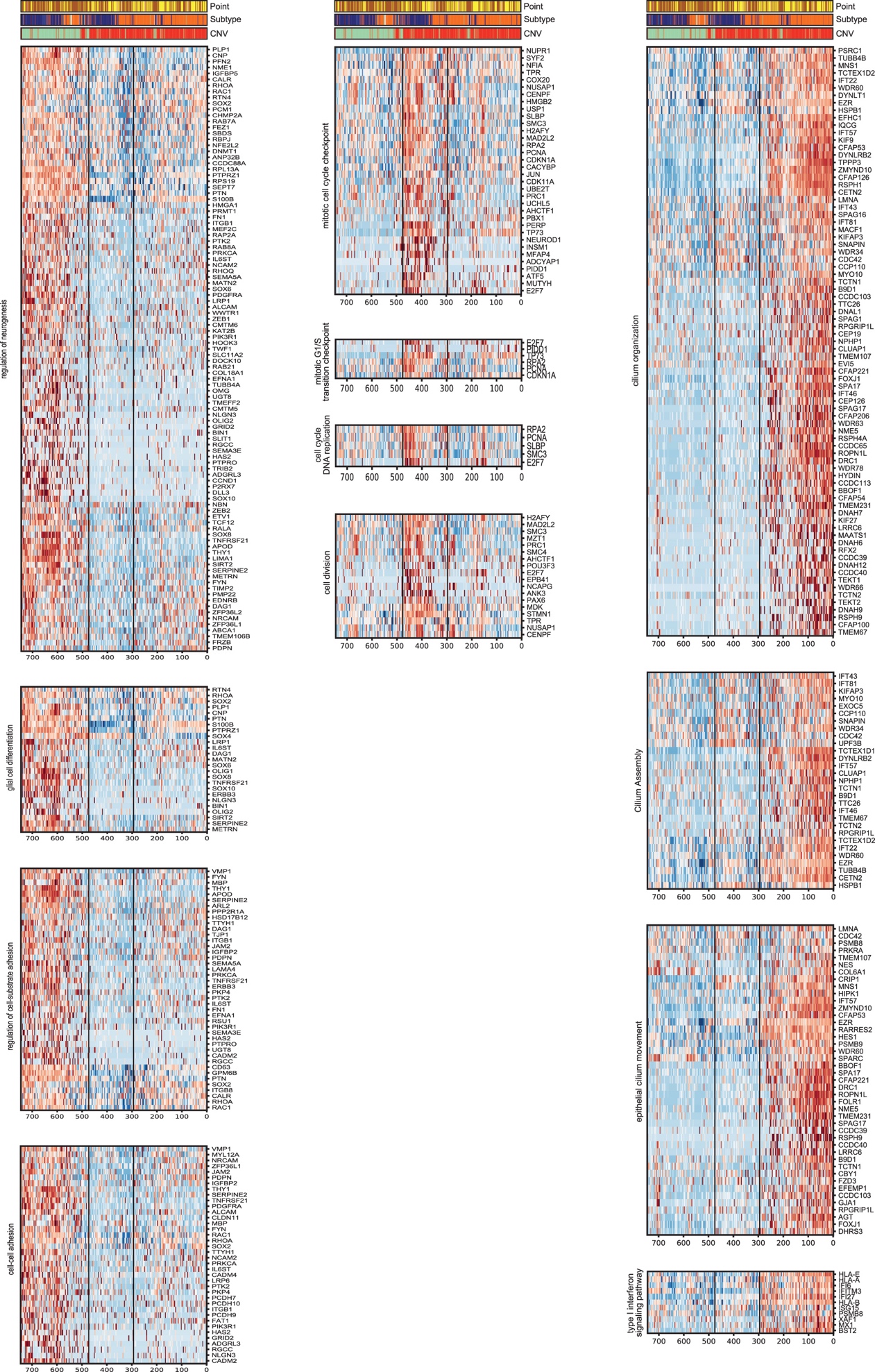
**

**Supplementary Fig. S12.** Gene expression heatmap of individual genes comprised in the GO terms listed in Figure 5G.

**
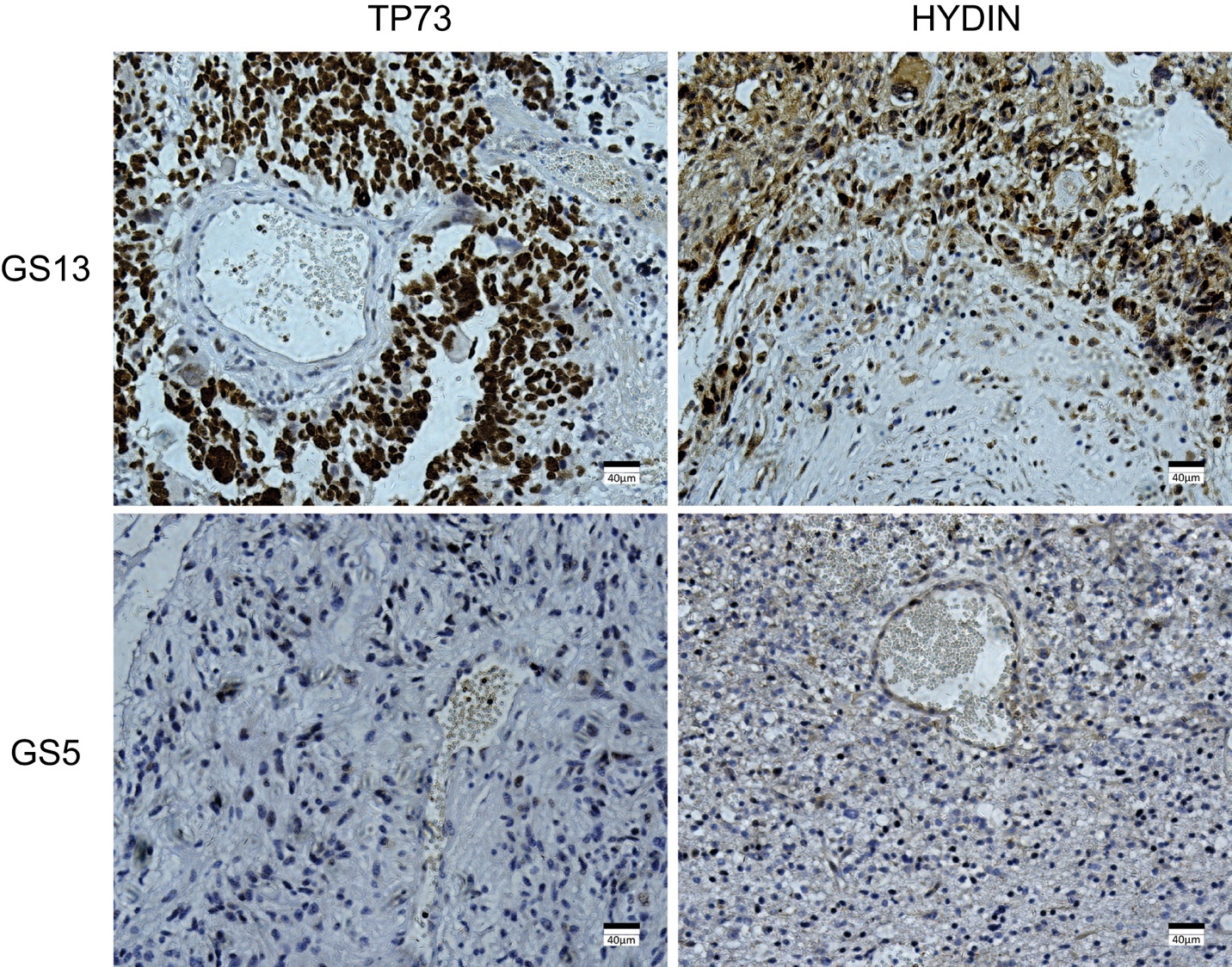
**

**Supplementary Fig. S13.** Immunohistochemistry staining image of *TP73* and *HYDIN* protein on paraffin slices of GS13 and GS5.

**Supplementary Fig. S14.** Expression level of full length *TP73* and *ΔN*-*TP73* isoform.

RT-qPCR of full length *TP73* and *ΔN*-*TP73* isoform. Both of them exists in GS13 cilium cells.

**
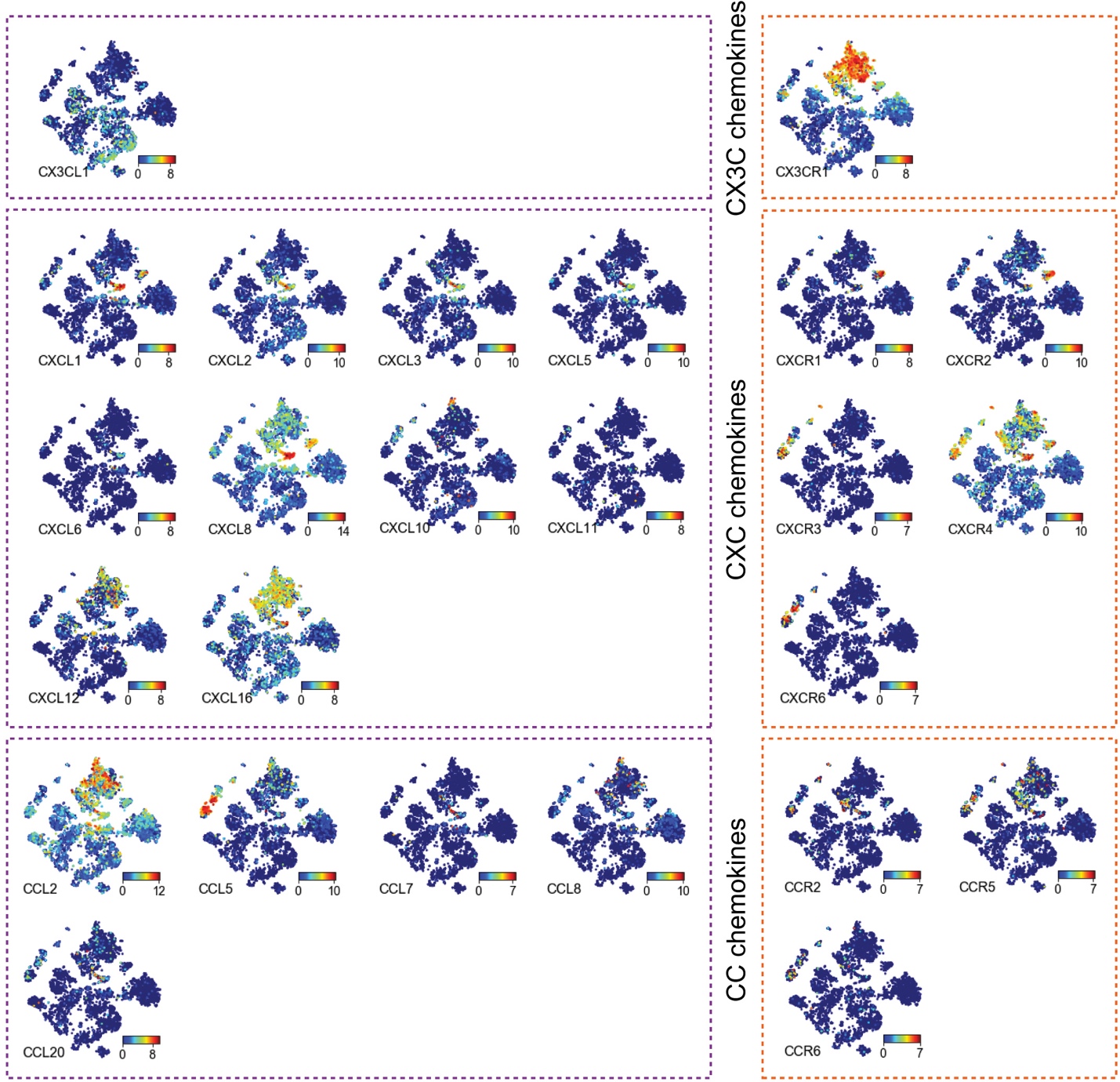
**

**Supplementary Fig. S15.** Cell source information of each patient in the pseudotime map from Fig. 6A**.**


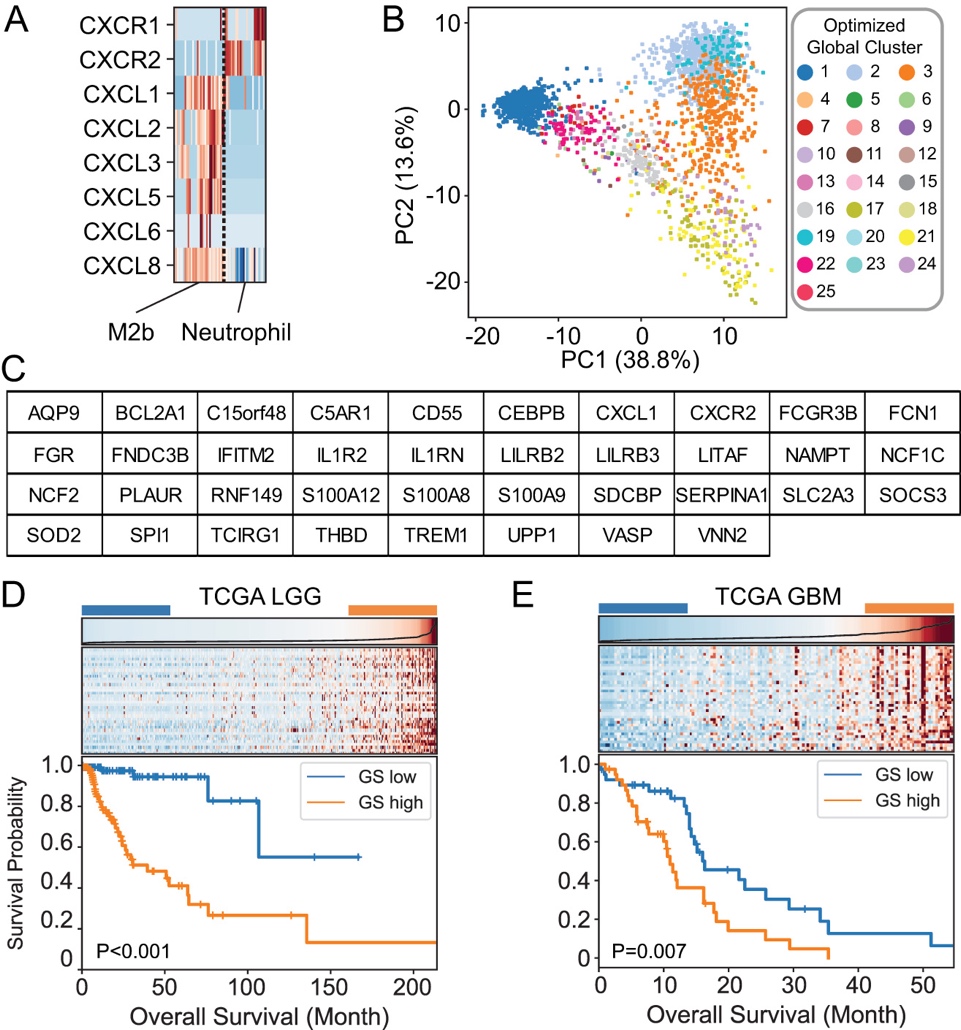


**Supplementary Fig. S16.** Characteristics of m2b macrophages and neutrophils and their potential in prognosis prediction using M2bNeu score

(A) Expression profile of chemotaxis related genes in M2b and neutrophil cells. (B) First 2 PCs of TME cell PCA analysis, using the same color code as (Fig. 2D) cell type color bar. M2b and neutrophils have similar characteristics in PC2. (C) M2bNeu gene list. (D-E) Prognosis prediction using the M2bNeu score. The scores were calculated with TCGA LGG/GBM bulk RNA-seq data. For each of these two groups of patients, samples in the upper quantiles (orange) of the ranked M2bNeu score showed a significantly worse prognosis than those in the lower quantiles (blue).

**
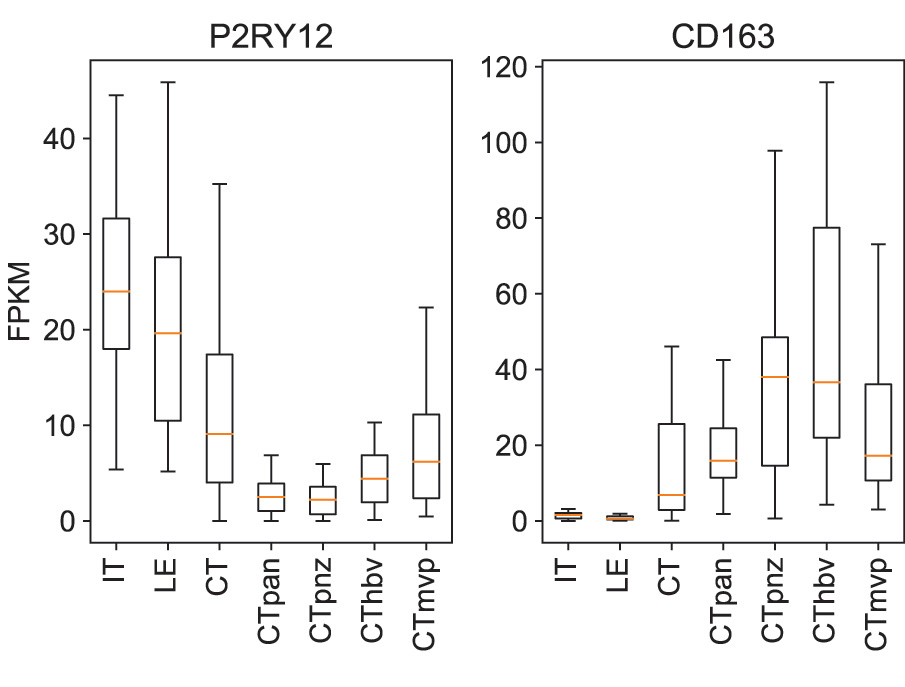
**

**Supplementary Fig. S17.** *P2RY12* and *CD163* expression in IVY GAP database.

5 major anatomic structures of glioblastoma: Leading Edge (LE), Infiltrating Tumor (IT), Cellular Tumor (CT), Pseudopalisading Cells Around Necrosis (pan), Peri-Necrotic Zone (pnz), Hyperplastic Blood Vessels (hbv), Microvascular Proliferation (mvp).

**
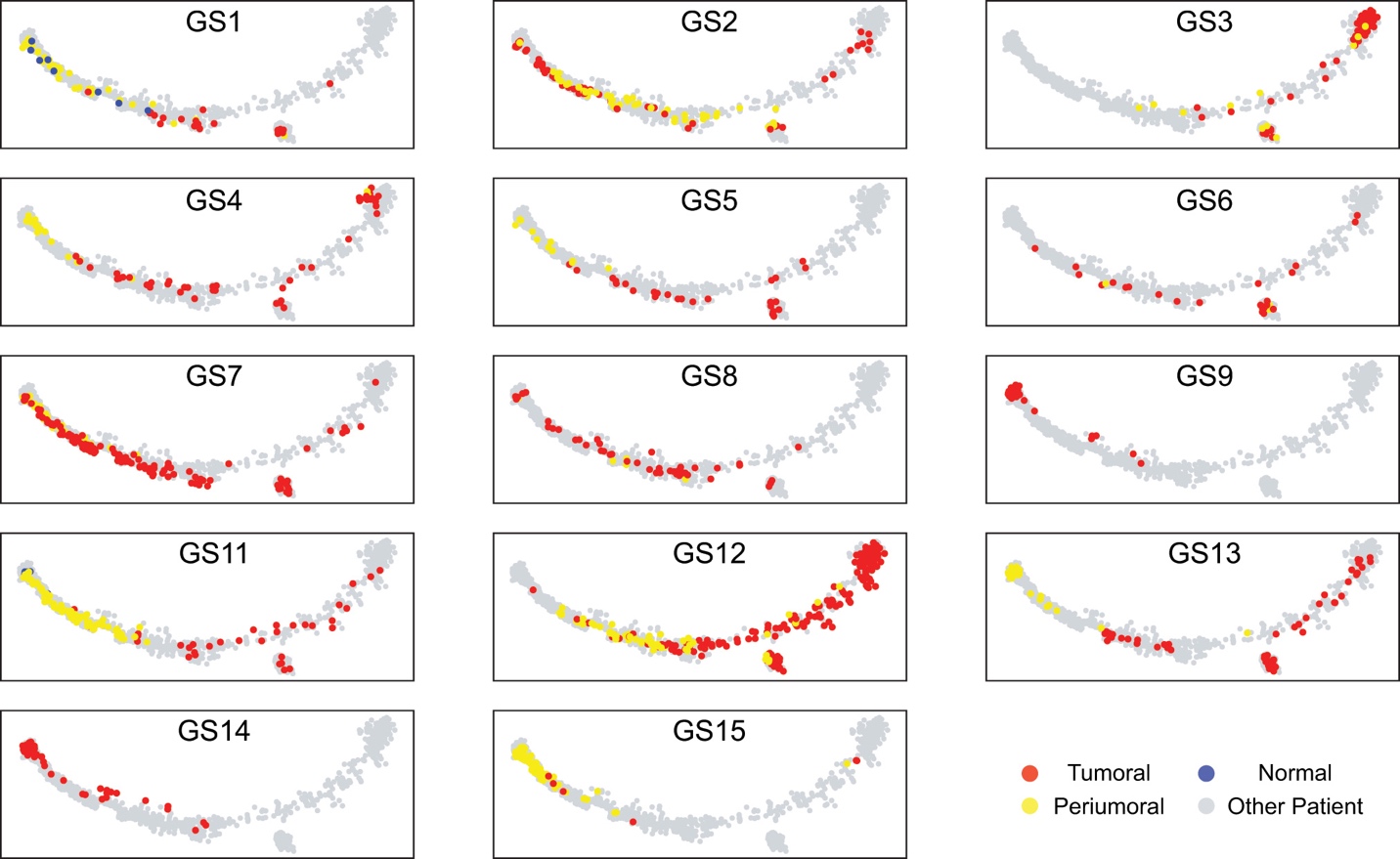
**

**Supplementary Fig. S18.** Cell source information of each patient in the pseudotime map from Fig. 6D.

# Supplementary Tables

**Supplementary Table 1.** Clinical information.

Clinical information of 14 patients and information of all biopsies.

**Supplementary Table 2.** Gene lists.

Marker genes of 25 clusters in the global t-SNE analysis and clustered marker genes related to Fig. 4F, 5F.
